# Highly efficient CRISPR editing enabled by magnetic nanoparticle delivery

**DOI:** 10.3389/fgeed.2026.1865675

**Published:** 2026-07-27

**Authors:** Inkyung Go, Seung Hwan Lee

**Affiliations:** Department of Life Science, Chung-Ang University, Seoul, Republic of Korea

**Keywords:** CRISPR, gene delivery, genome editing, highly efficient, magnetic nanoparticle

## Abstract

Efficient delivery of CRISPR components remains a major determinant of genome editing outcomes. In this study, we compared conventional lipofection with magnetic nanoparticle-assisted gene delivery (magnetofection) for CRISPR-mediated genome editing efficiency using SpCas9 and AsCas12a systems. Based on the average values obtained from multiple independent targets, lipofection resulted in relatively low indel efficiencies, with mean values of average 8.1%–12.47%. In contrast, magnetofection markedly enhanced genome editing outcomes, yielding average indel efficiencies of average 42.29%–45.04%, representing a substantial increase (3.39- and 5.56-fold, respectively) compared with lipofection. This enhancement was consistently observed across both SpCas9-and AsCas12a-mediated editing, indicating that the improved efficiency conferred by magnetic nanoparticle delivery is independent of the nuclease platform. Furthermore, the increased performance of magnetofection was reproducible across multiple genomic loci and cell lines and was also effective under RNP-based delivery conditions, demonstrating its robustness and reliability. In addition to indel-based genome disruption, magnetofection also significantly improved prime editing efficiency (13.95% on average) compared to lipofection (3.81% on average). Overall, our results demonstrate that magnetic nanoparticle-mediated delivery enables highly efficient and reproducible CRISPR genome editing, substantially outperforming conventional lipofection for both indel formation and prime editing. Magnetofection therefore represents a powerful and broadly applicable delivery strategy for next-generation genome editing applications.

## Introduction

CRISPR genome editing technology is an innovative and rapidly evolving platform that enables the reprogramming of target genetic information through RNA-guided recognition of genomic loci and the use of CRISPR–Cas effector endonucleases (Jinek et al., 2012; Cho et al., 2013; Cong et al., 2013). By utilizing guide RNAs to direct sequence-specific targeting, this technology allows for precise manipulation of genomic DNA and has become one of the most powerful tools for genetic engineering (Doudna and Charpentier, 2014; Pacesa et al., 2024). Currently, CRISPR-based technologies have evolved beyond the conventional approach of inducing insertions or deletions (indels) in target DNA sequences through guide RNA-mediated cleavage. More advanced forms of genome editing, including base editing and prime editing, have been developed (Komor et al., 2016; Gaudelli et al., 2017; Anzalone et al., 2019; Anzalone et al., 2020; Porto et al., 2020; Zhao et al., 2023). However, because these approaches rely on enzyme-mediated molecular processes, their overall editing efficiency often remains relatively low. In particular, genome editing strategies that modify target DNA without introducing double-strand breaks generally exhibit significantly lower efficiencies (Lee et al., 2018). Consequently, the development of efficient delivery systems capable of transporting genome editing components into appropriate cells and tissues is essential not only for research applications but also for therapeutic development in human patients (Li et al., 2023). As part of these efforts, a variety of delivery systems have been developed to achieve efficient delivery and expression of CRISPR components in diverse biological systems, including human-derived cells (Lino et al., 2018; Yip, 2020; Madigan et al., 2023; Tsuchida et al., 2024). These include non-viral delivery methods, such as lipofection and electroporation, as well as viral delivery systems primarily based on adeno-associated viruses (AAV). Lipofection methods enable relatively straightforward genome editing using commercially available cationic lipids; however, the overall efficiency of gene editing induced by this approach has been reported to be relatively limited (Hou et al., 2021; Chen et al., 2023; Herrera-Barrera et al., 2023; Holubowicz et al., 2025; Wu et al., 2026). Electroporation can achieve comparatively high editing efficiencies under stringent conditions involving electrical pulses, but it requires costly specialized equipment (Doman et al., 2022; Kim-Yip et al., 2024). In contrast, viral delivery systems for CRISPR components offer the advantage of enabling efficient genome editing in both *in vitro* and *in vivo* settings, although they carry the potential risk of unintended modification of host genomic DNA (Lino et al., 2018; Xu et al., 2019; Yip, 2020; Asmamaw Mengstie, 2022; She et al., 2023; Davis et al., 2024; Oh et al., 2024). Therefore, the development of efficient delivery strategies for CRISPR genome editing components across diverse biological systems is critically important for the successful implementation of genome editing technologies. According to recently published studies, the use of a simple and safe magnetic material-based delivery system has been shown to substantially enhance gene delivery efficiency across a wide range of biological systems (Rohiwal et al., 2020; Azadpour et al., 2023; Cho et al., 2024).

In this study, we analyzed the effects of applying a MAgnetic nanoparticle-based Gene Editing method (hereafter referred to as MAGE) on various CRISPR-mediated genome editing systems based on SpCas9 and AsCas12a, in comparison with conventional, commercially available LIpofectamine-based Gene Editing methods (hereafter referred to as LIGE). Continued exploration of gene delivery materials, including the MAGE technology evaluated in this study, is expected to contribute to the development of new and advanced genome editing technologies and to therapeutic applications through efficient *in vivo* delivery.

## Materials and methods

### Cell culture and maintenance

Human-derived cell lines (HEK293FT, K562, HeLa, and CuFi-1) were purchased from Invitrogen (R70007) and the American Type Culture Collection (CCL-243, CCL-2, and CRL-4013), respectively. HEK293FT, HeLa, and K562 cells were cultured in Dulbecco’s Modified Eagle Medium (DMEM, High Glucose, Pyruvate; Gibco, 11995073) supplemented with 10% fetal bovine serum (FBS, Premium Plus; Gibco, A5669701) and 1% penicillin–streptomycin (10,000 U/mL; Gibco, 15140122). CuFi-1 cells were cultured in serum-free Bronchial Epithelial Growth Medium (BEGM; Lonza, CC-3170), consisting of BEBM basal medium and SingleQuot additives, and supplemented with 50 μg/mL G-418. All cells were maintained at 37 °C in a CelCulture CO_2_ incubator (Esco Lifesciences, CCL-170B-8-FD) under 5% CO_2_ conditions.

### Construction of CRISPR expression plasmids and guide RNAs

Expression vectors for SpCas9 and AsCas12a were designed to operate under the control of a CMV promoter, and each guide RNA was expressed under a U6 promoter in an all-in-one vector format generated by in-house cloning. Target genomic sites were selected using CRISPR RGEN Tools (www.rgenome.net) (Supplementary Tables S1-S3). For insertion of target sequences, oligonucleotides were synthesized by Macrogen. In the SpCas9 system, the oligonucleotides were cloned into the SpCas9_sgRNA all-in-one vector using the restriction enzyme BsaI. In the AsCas12a system, the oligonucleotides were cloned into the AsCas12a_crRNA all-in-one vector using the restriction enzyme BsmBI. The plasmids pCMV_SpCas9 and pCMV_AsCas12a were extracted and purified from DH5α chemically competent *Escherichia coli* (enzynomics, CP011) using the AccuPrep® Nano-Plus Plasmid Mini Extraction Kit (BIONEER, K-3111) according to the manufacturer’s instructions. All plasmids were quantified using a NanoDrop One/OneC Microvolume UV–Vis Spectrophotometer (Thermo Scientific, ND-ONEC). For prime editing experiments, three expression vectors were prepared: a pegRNA plasmid expressed under a U6 promoter, a nicking guide RNA plasmid expressed under a U6 promoter, and a SpCas9(H840A)-RT plasmid expressed under a CMV promoter.

### Cell transfection

For transfection of adherent HEK293FT, HeLa, and CuFi-1 cells, cells were seeded in 24-well plates containing 400 μL of DMEM per well at a density of 0.5 × 10^5^ cells/mL so that approximately 70% confluency was reached on the day of transfection. Lipofection and magnetofection were compared under identical cellular conditions and CRISPR expression vector concentrations, with the delivery method as the variable (Lipofectamine 3000 for lipofection and PolyMAG Neo/MEGA Magnetic Plate for magnetofection). Cationic lipid-mediated delivery (lipofection) was performed using Lipofectamine 3000 Transfection Reagent (Invitrogen, L3000-015) and Opti-MEM, Reduced Serum Medium (Gibco, 31985070) according to the manufacturer’s protocol. For each well, 1 μg of plasmid DNA was diluted using 25 μL of Opti-MEM and subsequently mixed with 1 μL of P3000 Enhancer Reagent. Meanwhile, 1.5 μL of Lipofectamine 3000 was diluted using 25 μL of Opti-MEM and then combined with the DNA solution and incubated at room temperature for 15 min. The resulting DNA–lipid complexes were added dropwise to the cells, which were then incubated at 37 °C with 5% CO_2_. For suspension K562 cells, cells were dispensed into 24-well plates at 0.5 × 10^5^ cells/mL, and lipofection was immediately performed using the same transfection complex composition. Cells were harvested 48 h after transfection.

Magnetic nanoparticle-mediated delivery (magnetofection) was performed using PolyMAG Neo Magnetic Transfection Reagent (OZ Bioscience, PG61000) and a MEGA Magnetic Plate (OZ Bioscience, MF14000). For HEK293FT and HeLa cells, following the manufacturer’s protocol for adherent cells, 1 μg of plasmid DNA was diluted using 100 μL of Opti-MEM and subsequently mixed with 1 μL of PolyMAG Neo reagent in a 24-well plate format. The mixture was incubated for 15 min at room temperature to allow DNA–magnetic nanoparticle complex formation. Immediately after the addition of complexes to the cells, the culture plate was placed on a magnetic plate and incubated for 30 min. The plate was then removed from the magnetic plate and incubated further at 37 °C under 5% CO_2_ conditions. Magnetofection of suspension K562 cells was performed according to the manufacturer’s protocol for suspension cells. In a 24-well plate format, 1 μg of plasmid DNA was diluted using 50 μL of Opti-MEM, and 1 μL of PolyMAG Neo reagent was diluted using 50 μL of Opti-MEM. The two solutions were mixed and incubated at room temperature for 15–20 min to form DNA–magnetic nanoparticle complexes. At the same time, K562 cells were prepared at a density of 0.5 × 10^5^ cells/mL, and 30 μL of CombiMag reagent (OZ Bioscience, CM20100) was added per 1 mL of cell suspension, followed by incubation for 10–15 min. The cell suspension was then dispensed into a 24-well plate placed on a magnetic plate and incubated for 15 min, after which the prepared DNA–PolyMAG Neo complexes were added to the cells. The plate was incubated on the magnetic plate for an additional 15–20 min. The culture medium supernatant was carefully removed to avoid aspirating magnetically sedimented cells and replaced with fresh complete medium. The plate was then removed from the magnetic plate and incubated at 37 °C with 5% CO_2_. K562 cells were harvested 48 h after transfection.

For prime editing experiments, three major components were co-transfected: pegRNA (480 ng), nicking sgRNA (120 ng), and the CMV_SpCas9_RT prime editor plasmid (600 ng), totaling 1200 ng of DNA. Lipofection and magnetofection were performed according to the protocols described above, and both methods used the same total amount of DNA (1200 ng).

### CRISPR–Cas9 ribonucleoprotein (RNP) delivery

Cas9 protein was expressed in BL21(DE3) chemically competent *E. coli* (enzynomics, CP111) transformed with the expression vector (Kang et al., 2020). Cells were pre-cultured in LB broth (LPS Solution, LB-05) for 12–16 h and induced with IPTG (LPS Solution, IPTG025) at a final concentration of 1 mM for expression at 18 °C for 48 h or at 37 °C for 4 h. Cells were harvested by centrifugation at 5,100 × *g* for 10 min and resuspended in lysis buffer containing 20 mM Tris–HCl (pH 7.5–8.0), 300 mM NaCl, 10 mM β-mercaptoethanol, and 1% Triton X-100, supplemented with PMSF (Merck, 11359061001) to a final concentration of 100 mM. Cells were lysed using a Q500 Sonicator (Qsonica, Q500-110) at amplitude 30 for a total of 3 min with cycles of 10 son and 10 s off. The supernatant obtained after centrifugation was incubated with Ni-NTA resin (Qiagen, 30210) in a cold room for 1–1.5 h on a rocker. The resin-bound proteins were washed with wash buffer (20 mM Tris–HCl, pH 7.5–8.0, 500 mM NaCl). Bound proteins were sequentially eluted three times using elution buffer containing 20 mM Tris–HCl (pH 7.5–8.0), 300 mM NaCl, and 200 mM imidazole (LPS Solution, CBM01). The eluted fractions were collected separately and designated as E1, E2, and E3. Protein fractions were buffer-exchanged using Amicon® Ultra Centrifugal Filters (100 kDa MWCO; Merck, UFC510024) into storage buffer containing 10 mM HEPES-KOH (pH 7.5), 250 mM KCl, 1 mM MgCl_2_, 0.1 mM EDTA, 7 mM β-mercaptoethanol, and 20% glycerol. Protein concentration was measured using a NanoDrop One/OneC spectrophotometer, and aliquots of 10–20 μL were stored at −80 °C.

The guide RNA was generated by *in vitro* transcription (IVT). Briefly, extension PCR was performed to prepare the sgRNA template DNA. The PCR reaction mixture consisted of 2.5 μL of primer F (100 pmol), 2.5 μL of primer R (100 pmol), 25 μL of KOD One PCR Master Mix-Blue (TOYOBO, KMM-201), and 21 μL of distilled water. The annealing condition was determined based on the Tm value of the overlapping region between the primers. PCR conditions consisted of an initial denaturation at 98 °C for 2 min, followed by 35 cycles of denaturation at 98 °C for 10 s, annealing for 5 s, and extension at 68 °C for 5 s, with a final extension at 68 °C for 5 min. The amplified PCR product was purified using a QIAquick PCR Purification Kit (Qiagen, 28106). The purified DNA template (3–5 μg) was then used for *in vitro* transcription.

The transcription reaction was prepared by mixing 3–5 μg of DNA template, 4 μL each of the 100 mM rNTP set (Bioneer, D-3003), 28 μL of MgCl_2_, 10 μL of 10× buffer, 5 μL of T7 RNA polymerase (New England Biolabs, M0251L), 1 μL of RNase inhibitor, 1 μL of DTT (100 mM), and 29 μL of UltraPure DEPC-Treated Water (Invitrogen, 750023). The reaction mixture was incubated overnight at 37 °C in an incubator (Jeiotech, IB-01E). To remove the remaining DNA template, 1 μL of RNase-free DNase I (New England Biolabs, M0303L) was added, followed by an additional incubation at 37 °C for 30 min. The synthesized RNA was then purified using a QIAquick PCR Purification Kit (Qiagen, 28106), eluted in 50 μL of DEPC-treated water, and used for subsequent experiments. RNP complexes were formed by incubating 500 ng of Cas9 protein with gRNA at room temperature (approximately 20 °C–25 °C) for 10–20 min. The molar ratios of Cas9:gRNA were set to 1:1, 1:2, 1:4, and 1:8, corresponding to 125.2, 250.5, 501, and 1002 ng of gRNA, respectively, for 500 ng Cas9 protein. The assembled RNP complexes were delivered into cells using either lipofection or magnetofection, as described above.

### Genomic DNA extraction and PCR amplification

Cells were harvested 48–72 h after transfection. Genomic DNA was extracted using the DNeasy Blood & Tissue Kit (Qiagen, 69506) according to the manufacturer’s protocol. Target-specific primers (Supplementary Tables S4) for amplification of each genomic locus were designed using SnapGene software. PCR amplification was performed using KOD One PCR Master Mix-Blue (TOYOBO, KMM-201). Reactions were carried out in a total volume of 21.6 μL containing 1 μL of template DNA and primers at a concentration of 10 pmol each. PCR conditions consisted of an initial denaturation at 98 °C for 2 min, followed by 35 cycles of denaturation at 98 °C for 10 s, annealing at 59 °C for 5 s, and extension at 68 °C for 5 s, with a final extension at 68 °C for 5 min.

### Preparation of next-generation sequencing libraries and data analysis

PCR amplification for next-generation sequencing was performed in three sequential steps. In the first PCR, the target genomic region (∼700–800 bp) was amplified using target-specific primers (Supplementary Tables S4) and KOD One PCR Master Mix-Blue (TOYOBO, KMM-201). The reaction was performed in a total volume of 21.6 μL containing 1 μL of genomic DNA and primers at a concentration of 10 pmol each. PCR conditions consisted of an initial denaturation at 98 °C for 2 min, followed by 35 cycles of denaturation at 98 °C for 10 s, annealing at 59 °C for 5 s, and extension at 68 °C for 5 s, with a final extension at 68 °C for 5 min. The second PCR was performed as a nested PCR using 1 μL of the first PCR product as the template, thereby attaching sequencing adapters to a ∼200 bp amplicon. The reaction composition for the second PCR was identical to that of the first PCR, consisting of a total reaction volume of 21.6 μL containing 1 μL of the first PCR product and primers at 10 pmol each. The PCR conditions were also identical to those of the first PCR: an initial denaturation at 98 °C for 2 min, followed by 35 cycles of denaturation at 98 °C for 10 s, annealing at 59 °C for 5 s, and extension at 68 °C for 5 s, with a final extension at 68 °C for 5 min. In the third PCR, 1 μL of the second PCR product was used as the template to attach unique forward and reverse indices to each sample. The third PCR was also performed in a total reaction volume of 21.6 μL with primers at 10 pmol each. PCR conditions consisted of an initial denaturation at 98 °C for 2 min, followed by 35 cycles of denaturation at 98 °C for 10 s, annealing at 60 °C for 5 s, and extension at 68 °C for 5 s, with a final extension at 68 °C for 5 min. The three PCR steps were performed sequentially without intermediate purification. The final PCR products were purified using the QIAquick PCR Purification Kit (Qiagen, 28106) and quantified using a NanoDrop One/OneC Microvolume UV-Vis Spectrophotometer (Thermo Scientific, ND-ONEC). The purified PCR products were subjected to paired-end sequencing on an Illumina MiSeq or MiniSeq platform. NGS data were analyzed using the web-based analysis tool Cas-Analyzer from CRISPR RGEN Tools (www.rgenome.net). Using this method, both indel frequencies and precise prime editing efficiencies were quantified. Indel frequencies were calculated by comparing edited samples with negative controls, while prime editing efficiency was determined by detecting the intended nucleotide substitutions.

### Statistical analysis

All experiments were performed with three independent biological replicates. Data are presented as the mean values. Statistical significance was determined using two-way ANOVA. A *p* value of less than 0.05 was considered statistically significant and is indicated by asterisks in the figures.

### GFP fluorescence imaging and quantification

To assess plasmid delivery efficiency, GFP fluorescence was analyzed in HEK293FT cells following delivery of the GFP-expressing CRISPR plasmid pCAG-SpCas9-GFP-U6-gRNA. The plasmid was a gift from Jizhong Zou and obtained from Addgene (Addgene plasmid #79144). HEK293FT cells were cultured and seeded in Costar® 24-well Clear TC-treated Multiple Well Plates (Corning, cat. no. 3524) under the same conditions described above. LIGE and MAGE were performed using the same transfection conditions described above, with the delivery method as the only variable. The following experimental groups were prepared: untreated control, LIGE reagent only, MAGE reagent only, LIGE + GFP plasmid, MAGE + GFP plasmid, and GFP plasmid only. For GFP-containing delivery groups, 1 µg of pCAG-SpCas9-GFP-U6-gRNA plasmid DNA per well was delivered using either LIGE or MAGE. The LIGE-only and MAGE-only groups received the corresponding delivery reagents without plasmid DNA, whereas the GFP-only group received plasmid DNA without delivery reagent. Untreated cells received neither plasmid DNA nor delivery reagent. No medium replacement was performed after transfection, and cells were incubated for 48 h after transfection at 37 °C under 5% CO_2_. At 48 h post-transfection, GFP fluorescence images were acquired using an Agilent BioTek Cytation 5 Cell Imaging Only Reader (Agilent BioTek, CYT5PW) with a standard GFP fluorescence channel. Images were acquired using the same Gen5 acquisition protocol across all experimental groups. Total GFP fluorescence was quantified using Agilent BioTek Gen5 software. For quantitative comparison, the total GFP fluorescence intensity from each experimental group was measured and normalized to the mean value of the LIGE + GFP plasmid group, which was set to 100%. The normalized values are presented as normalized total GFP fluorescence (%). Data are shown as the mean ± standard error from three independent transfection replicates.

### Cell viability assay

Cell viability after LIGE- and MAGE-mediated delivery of CRISPR plasmids was evaluated using a trypan blue exclusion assay. HEK293FT cells were seeded in 24-well plates containing 400 μL of complete DMEM per well at a density of 0.5 × 10^5^ cells/mL so that approximately 70% confluency was reached on the day of transfection. Cells were assigned to the following groups: untreated control, LIGE reagent only, MAGE reagent only, LIGE + GFP plasmid, MAGE + GFP plasmid, and GFP plasmid only. For plasmid-containing groups, 1 μg of the GFP-expressing CRISPR plasmid pCAG-SpCas9-GFP-U6-gRNA per well was used. LIGE and MAGE treatments were performed under the same conditions described above using Lipofectamine 3000 and PolyMAG Neo with a MEGA Magnetic Plate, respectively. Reagent-only control groups were treated with the corresponding delivery reagent in the absence of plasmid DNA, whereas the plasmid-only group received plasmid DNA without transfection reagent. At 48 h after transfection, cells were harvested for viability analysis. To include both floating and adherent cells, the culture medium from each well was first collected into a tube. The wells were gently washed with phosphate-buffered saline (D-PBS, 1X; Welgene, LB001-02-AA), and adherent cells were detached using 0.25% Trypsin-EDTA (phenol red; Gibco, 25200072). Trypsinization was neutralized by adding complete culture medium, and the detached cells were combined with the previously collected culture medium. The pooled cell suspension was gently pipetted to obtain a single-cell suspension. The cell suspension was mixed with 0.4% trypan blue solution supplied with the EVE™ Slide (NanoEnTek, Seoul, Republic of Korea; EVS-050) at a 1:1 ratio. The mixture was loaded onto a disposable cell counting slide, and viable and non-viable cells were counted using an EVE™ Automated Cell Counter (NanoEnTek, Seoul, Republic of Korea; EVE-MC) according to the manufacturer’s instructions. Data are presented as the mean ± standard error of the mean from three independent biological replicates. Statistical significance was analyzed using one-way ANOVA followed by a multiple-comparison test, and *p*-values less than 0.05 were considered statistically significant.

## Results

### Magnetic nanoparticle-mediated delivery of CRISPR expression vectors significantly enhances SpCas9-and AsCas12a-mediated genome editing efficiency

**FIGURE 1:**
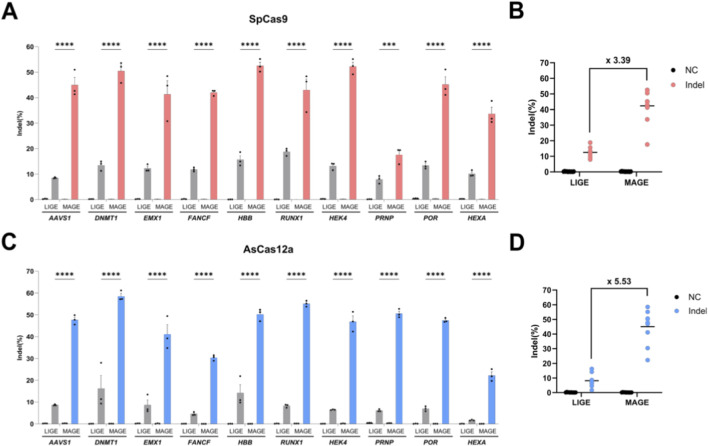
High-efficiency SpCas9-and AsCas12a-mediated genome editing by magnetic nanoparticle delivery. **(A,C)** Comparison of indel frequencies across multiple genomic loci (*AAVS1, DNMT1, EMX1, FANCF, HBB, RUNX1, HEK4, PRNP, POR*, and *HEXA*) following delivery of SpCas9 or AsCas12a effectors into HEK293FT cells using lipofection or magnetofection. **(B,D)** Average indel frequencies across all target loci induced by SpCas9 or AsCas12a effectors delivered *via* lipofection or magnetofection, corresponding to the experiments shown in panels **(A,C)**. Genome editing efficiency was determined by PCR amplification of the target genomic regions 48 h after transfection, followed by next-generation sequencing (NGS) analysis. Indel frequencies were calculated using Cas-Analyzer. Data are presented as the mean ± standard error from three independent biological replicates. Statistical significance was evaluated using two-way ANOVA (ns: not significant, *p ≤ 0.05, **p ≤ 0.01, ***p ≤ 0.001, ****p ≤ 0.0001).

To evaluate the impact of delivery methods on CRISPR–Cas-mediated genome editing, we compared conventional lipofection–based genome editing (LIGE) with magnetic nanoparticle-based genome editing (MAGE) in a human-derived cell line (HEK293FT) using SpCas9 (Cho et al., 2013) and AsCas12a (Zhang et al., 2021) systems (Figure 1). Multiple endogenous genomic loci, including *AAVS1, DNMT1, EMX1, FANCF, HBB, RUNX1, HEK4, PRNP, POR,* and *HEXA*, were targeted (Supplementary Figure S1) to enable a direct comparison of the two delivery methods under identical transfection conditions, with the only variable being the delivery reagent (LIGE vs. MAGE components). Targeted deep sequencing performed 48 h after transfection revealed that lipofection yielded relatively low genome editing efficiencies, with average indel frequencies of approximately 12.47% for SpCas9 (Figures 1A,B; Supplementary Figure S2; Supplementary Tables S1, 2) and 8.1% for AsCas12a (Figures 1C,D; Supplementary Figure S2; Supplementary Tables S1, 2). In contrast, magnetofection increased the average indel efficiency to approximately 42.29% for SpCas9 (Figures 1A,B; Supplementary Figure S2; Supplementary Tables S1, 2) and 45.04% for AsCas12a (Figures 1C,D; Supplementary Figure S2; Supplementary Tables S1, 2) under the same cellular and CRISPR expression vector conditions, representing an average 3.39-fold and 5.56-fold improvements, respectively (Figures 1B,D). Importantly, this enhancement in editing efficiency was consistently observed for both SpCas9 and AsCas12a, indicating that the efficiency improvement mediated by magnetofection is not dependent on a specific nuclease system. When averaged across all genomic targets, magnetofection produced significantly higher editing efficiencies compared with lipofection, demonstrating enhanced delivery efficiency and strong experimental reproducibility without severe cell toxicity (Supplementary Figure S3).

### Magnetic nanoparticle-based delivery induces high genome editing efficiency in diverse human cell lines

**FIGURE 2:**
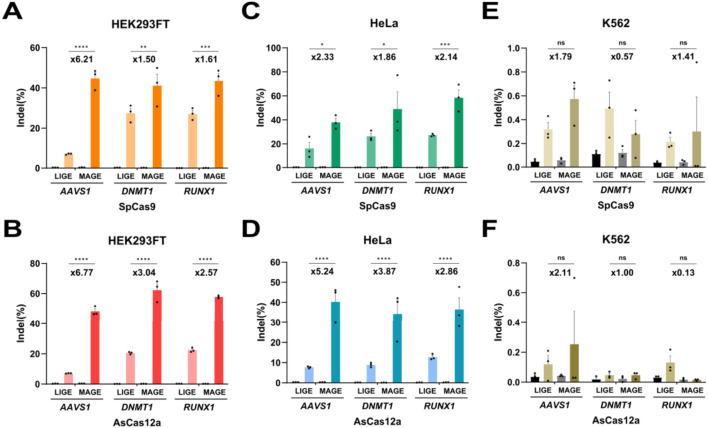
Magnetic nanoparticle-mediated delivery enables highly efficient genome editing across diverse cell lines. **(A,B)** Comparison of indel induction efficiencies following delivery of SpCas9 or AsCas12a effectors *via* lipofection or magnetofection in HEK293FT cells. **(C,D)** Comparison of indel induction efficiencies following delivery of SpCas9 or AsCas12a effectors *via* lipofection or magnetofection in HeLa cells. **(E,F)** Comparison of indel induction efficiencies following delivery of SpCas9 or AsCas12a effectors *via* lipofection or magnetofection in K562 cells. All editing efficiencies were quantified using next-generation sequencing (NGS) and are presented as the mean ± standard error from three independent biological replicates. Statistical significance was evaluated using two-way ANOVA (ns: not significant, *p ≤ 0.05, **p ≤ 0.01, ***p ≤ 0.001, ****p ≤ 0.0001).

To determine whether the MAGE-based improvement in editing efficiency is generalizable across different cellular contexts, we performed the same experiments in HEK293FT, HeLa, and K562 cell lines (Figure 2). In adherent cell lines (HEK293FT and HeLa), genome editing efficiencies mediated by SpCas9 and AsCas12a were significantly higher with magnetofection compared with lipofection (Figures 2A–D). The magnitude of efficiency improvement was comparable between the two adherent cell types, suggesting that MAGE is broadly applicable across multiple adherent human cell types. To further evaluate potential gene editing applicability, we tested magnetofection in Cufi-1 cells, a cystic fibrosis disease model cell line (Supplementary Figure S4). Genome editing experiments targeting multiple genomic loci showed that magnetofection consistently produced higher indel frequencies than lipofection across all tested targets. These results demonstrate that magnetofection can be effectively applied in clinically relevant disease model cells. In contrast, genome editing efficiencies mediated by SpCas9 and AsCas12a in the suspension cell line K562 did not show a significant increase with magnetofection compared to lipofection (Figures 2E,F).

### Magnetic nanoparticle-mediated delivery significantly enhances prime editing efficiency

**FIGURE 3:**
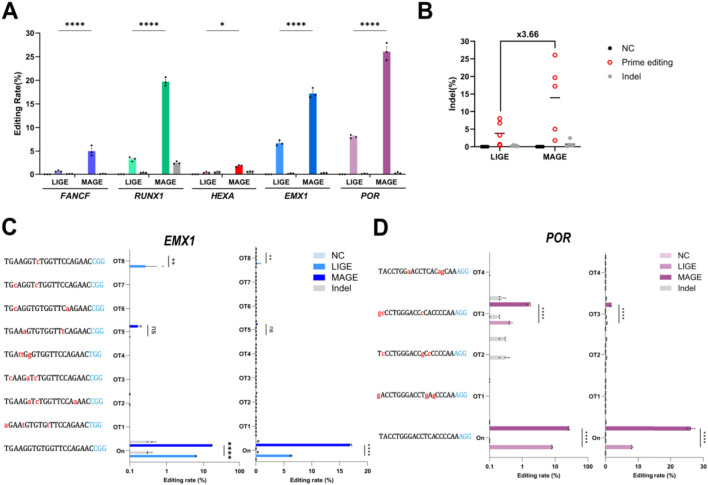
High-efficiency prime editing by magnetic nanoparticle delivery. **(A)** Comparison of prime editing efficiencies following delivery of prime editor components *via* lipofection or magnetofection in HEK293FT cells. Equal amounts of CMV_SpCas9-RT, pegRNA, and nicking sgRNA expression vectors were delivered using either lipofection or magnetofection. **(B)** Average frequencies of prime editing and unintended indels induced at multiple genomic loci (*FANCF, RUNX1, HEXA, EMX1*, and *POR*) following delivery of prime editor components by lipofection or magnetofection, corresponding to the experiment shown in panel **(A)**. **(C,D)** In silico-predicted off-target genome editing analysis following prime editing at the *EMX1* and *POR* target loci. PAM sequences are shown in blue. Guide RNA-DNA mismatches are highlighted in red. Prime editing and indel frequencies (%) were quantified using next-generation sequencing (NGS) of the target loci and are presented as the mean ± standard error from three independent biological replicates. Statistical significance was determined using two-way ANOVA (ns: not significant, *p ≤ 0.05, **p ≤ 0.01, ***p ≤ 0.001, ****p ≤ 0.0001).

Prime editing requires the simultaneous delivery of multiple components, including the prime editor protein, prime editing guide RNA (pegRNA), and nicking guide RNA (ngRNA), making editing efficiency highly dependent on the intracellular delivery efficiency of these components (Anzalone et al., 2019; Doman et al., 2022; Zhao et al., 2023). To evaluate the effect of magnetofection on prime editing, equal amounts of prime editor plasmid, pegRNA, and ngRNA were delivered into HEK293FT cells using either lipofection or magnetofection (Figure 3; Supplementary Table S3). In lipofection-treated cells, prime editing efficiencies were generally low, with an average efficiency of 3.81% (Figures 3A,B). In contrast, magnetofection achieved precise editing efficiencies of approximately 13.95% across multiple genomic targets, including *FANCF, RUNX1, HEXA, EMX1*, and *POR*. In silico-based off-target analysis performed for *EMX1* and *POR* targets indicated that magnetofection did not substantially increase off-target editing compared with lipofection (Figures 3C,D). Importantly, a slight increase in prime editing events was observed at certain off-target sites (Figure 3D). Compared with conventional CRISPR-Cas9 targeting (Supplementary Figure S3E, F), the off-target activity of the prime editor showed a distinct dependence on both the number and position of mismatches within the protospacer sequence. Taken together, these findings suggest that enhanced delivery efficiency, when combined with additional strategies designed to suppress off-target activity, may further improve the editing efficiency of complex systems, such as prime editing, while maintaining target specificity.

### Magnetic nanoparticle-mediated delivery of CRISPR RNP complexes produces higher genome editing efficiency compared with lipofection

**FIGURE 4:**
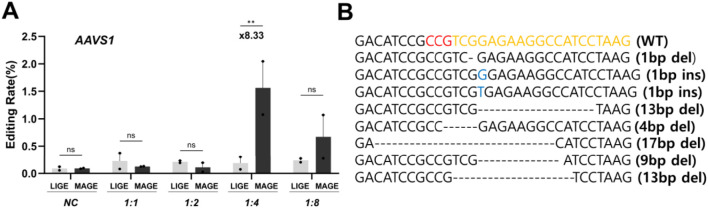
High-efficiency RNP-based genome editing by magnetic nanoparticle delivery. **(A)** Comparison of indel induction efficiencies following delivery of Cas9–gRNA ribonucleoprotein (RNP) complexes *via* lipofection or magnetofection in HEK293FT cells at different Cas9:gRNA molar ratios (1:1, 1:2, 1:4, and 1:8). **(B)** Analysis of representative indel patterns generated by Cas9–gRNA RNP delivery. PAM sequences are shown in red. Inserted sequences are highlighted in blue and deleted sequences are shown as dashed lines. Indel frequencies were quantified by next-generation sequencing (NGS) 48 h after RNP complex delivery. Data are presented as the mean ± standard error from two independent biological replicates. Statistical significance was evaluated using two-way ANOVA (ns: not significant, *p ≤ 0.05, **p ≤ 0.01, ***p ≤ 0.001, ****p ≤ 0.0001).

In addition to plasmid-based delivery of CRISPR components, we evaluated the delivery efficiency of preassembled Cas9–guideRNA ribonucleoprotein (RNP) complexes, which are widely used for genome editing applications (Figure 4). Cas9:gRNA molar ratios (1:1, 1:2, 1:4, and 1:8) were tested, and the resulting RNP complexes were delivered into HEK293FT cells using either lipofection or magnetofection. Under specific molar ratio conditions (1:4), magnetofection produced significantly higher indel frequencies (average value 1.56%) compared with lipofection (average value 0.19%) (Figure 4A). Analysis of indel mutation patterns revealed no significant differences in the mutation spectra between the two delivery methods, indicating that magnetofection enhances delivery efficiency without altering the underlying DNA repair pathways (Figure 4B; Supplementary Figure S1). These results demonstrate that magnetic nanoparticle–based delivery can be effectively applied not only to plasmid-based CRISPR systems but also to RNP-based genome editing platforms.

## Discussion

This study demonstrates that magnetic nanoparticle-mediated delivery can consistently enhance genome editing efficiency across multiple CRISPR systems. Original study shows that magnetic nanoparticles can be efficiently concentrated at the cell surface under an external magnetic field, thereby increasing the frequency of physical interactions with the plasma membrane and facilitating cellular uptake (Cho et al., 2024). This property enables efficient intracellular delivery even at relatively low concentrations of editing components, which may ultimately contribute to the observed increase in genome editing efficiency. In this study, we achieved efficient intracellular delivery by immobilizing either negatively charged plasmid DNA encoding CRISPR components or guide RNA–protein ribonucleoprotein (RNP) complexes onto positively charged magnetic particles. The resulting particle–cargo complexes were subsequently deposited onto cultured cells under an external magnetic field, thereby markedly enhancing cellular delivery efficiency. We systematically expanded the application of previously established magnetic nanoparticle-based delivery platforms to a diverse range of CRISPR modules, technologies, and methodological frameworks, accompanied by comprehensive evaluations of their performance, efficiency, and functional characteristics. Importantly, the reproducible results obtained across multiple genomic targets and cell types underscore the robustness and versatility of this delivery platform, supporting its potential as a broadly applicable tool for genome engineering applications. Notably, comparable improvements in editing efficiency were observed for both SpCas9 and AsCas12a, suggesting that the beneficial effects of magnetofection are unlikely to arise from enhanced nuclease activity itself, but rather from improved intracellular delivery and accumulation of CRISPR components. Collectively, these findings highlight intracellular delivery as a critical determinant of genome editing efficiency and demonstrate that optimization of delivery strategies alone can substantially improve CRISPR-mediated genome engineering outcomes. The efficiency improvements mediated by magnetofection were reproducibly observed not only in HEK293FT cells but also in HeLa cells and disease model cell lines, indicating that this delivery strategy possesses a degree of broad applicability across different cellular contexts. Conventional lipofection often exhibits substantial variability in delivery efficiency depending on the cell type and frequently shows reduced performance in cell types of particular therapeutic relevance. In this regard, magnetic nanoparticle-based delivery represents a promising platform capable of achieving consistently high genome editing efficiencies across diverse cellular environments. However, owing to the intrinsic characteristics of the magnetofection approach, no significant improvement was observed in suspension cell lines, indicating that certain limitations remain for its application across all cell types. Prime editing represents a complex genome editing platform requiring the simultaneous delivery of multiple components, including the prime editor protein, pegRNA, and nicking sgRNA. As a result, its editing efficiency is highly dependent on the intracellular delivery efficiency of these components. The observation that magnetofection significantly enhanced prime editing efficiency in this study highlights the critical importance of delivery optimization for the practical implementation of next-generation precision genome editing technologies. Importantly, the increase in editing efficiency was not accompanied by a substantial increase in unintended indel formation, suggesting that the improved delivery efficiency does not inherently compromise editing fidelity. This observation indicates that further optimization strategies, such as combining magnetofection with additional approaches designed to enhance target specificity, could further improve the performance of precision genome editing platforms. In addition, our finding that magnetofection effectively enhanced delivery efficiency in RNP-based CRISPR systems demonstrates that this delivery strategy is applicable not only to DNA-based expression systems, but also to protein–RNA ribonucleoprotein complexes. RNP-based delivery offers several advantages, including transient nuclease activity, reduced off-target risk, and elimination of insertional mutagenesis associated with DNA delivery, making it an attractive strategy for therapeutic genome editing applications. Considering these advantages, magnetic nanoparticle-mediated delivery may provide a versatile platform with potential for future genome editing, including *in vivo* applications.

Taken together, magnetic nanoparticle-mediated delivery provides superior efficiency and reproducibility compared with conventional lipofection and can function as a versatile delivery platform applicable to plasmid-based CRISPR systems, prime editing, and RNP-based genome editing approaches. The implementation of such delivery strategies is expected to play a critical role in improving the efficiency of next-generation genome editing technologies while simultaneously expanding their potential for biological applications.

## Data Availability

The datasets presented in this study can be found in online repositories. The names of the repository/repositories and accession number(s) can be found in the article/Supplementary Material.

## References

[B1] Anzalone A. V. Randolph P. B. Davis J. R. Sousa A. A. Koblan L. W. Levy J. M. (2019). Search-and-replace genome editing without double-strand breaks or donor DNA. Nature 576, 149–157. 10.1038/s41586-019-1711-4 31634902 PMC6907074

[B2] Anzalone A. V. Koblan L. W. Liu D. R. (2020). Genome editing with CRISPR-Cas nucleases, base editors, transposases and prime. Nat. Biotechnol. 38, 824–844. 10.1038/s41587-020-0561-9 32572269

[B3] Asmamaw Mengstie M. (2022). Viral vectors for the *in vivo* delivery of CRISPR components: advances and challenges. Front. Bioeng. Biotechnol. 10, 895713. 10.3389/fbioe.2022.895713 35646852 PMC9133430

[B4] Azadpour B. Aharipour N. Paryab A. Omid H. Abdollahi S. Madaah Hosseini H. (2023). Magnetically-assisted viral transduction (magnetofection) medical applications: an update. Biomater. Adv. 154, 213657. 10.1016/j.bioadv.2023.213657 37844415

[B5] Chen Z. Kelly K. Cheng H. Dong X. Hedger A. K. Li L. (2023). *In vivo* prime editing by lipid nanoparticle Co-delivery of chemically modified pegRNA and prime editor mRNA. Gen. Biotechnol. 2, 490–502. 10.1089/genbio.2023.0045 39850578 PMC11756591

[B6] Cho S. W. Kim S. Kim J. M. Kim J. S. (2013). Targeted genome engineering in human cells with the Cas9 RNA-Guided endonuclease. Nat. Biotechnol. 31, 230–232. 10.1038/nbt.2507 23360966

[B7] Cho H. Y. Yoo M. Pongkulapa T. Rabie H. Muotri A. R. Yin P. T. (2024). Magnetic nanoparticle-assisted non-viral CRISPR-Cas9 for enhanced genome editing to treat Rett syndrome. Adv. Sci. (Weinh) 11, e2306432. 10.1002/advs.202306432 38647391 PMC11200027

[B8] Cong L. Ran F. A. Cox D. Lin S. Barretto R. Habib N. (2013). Multiplex genome engineering using CRISPR/Cas systems. Science 339, 819–823. 10.1126/science.1231143 23287718 PMC3795411

[B9] Davis J. R. Banskota S. Levy J. M. Newby G. A. Wang X. Anzalone A. V. (2024). Efficient prime editing in mouse brain, liver and heart with dual AAVs. Nat. Biotechnol. 42, 253–264. 10.1038/s41587-023-01758-z 37142705 PMC10869272

[B10] Doman J. L. Sousa A. A. Randolph P. B. Chen P. J. Liu D. R. (2022). Designing and executing prime editing experiments in mammalian cells. Nat. Protoc. 17, 2431–2468. 10.1038/s41596-022-00724-4 35941224 PMC9799714

[B11] Doudna J. A. Charpentier E. (2014). Genome editing. The new frontier of genome engineering with CRISPR-Cas9. Science 346, 1258096. 10.1126/science.1258096 25430774

[B12] Gaudelli N. M. Komor A. C. Rees H. A. Packer M. S. Badran A. H. Bryson D. I. (2017). Programmable base editing of A*T to G*C in genomic DNA without DNA cleavage. Nature 551, 464–471. 10.1038/nature24644 29160308 PMC5726555

[B13] Herrera-Barrera M. Gautam M. Lokras A. Vlasova K. Foged C. Sahay G. (2023). Lipid nanoparticle-enabled intracellular delivery of prime editors. AAPS J. 25, 65. 10.1208/s12248-023-00833-2 37380935 PMC13198972

[B14] Holubowicz R. Du S. W. Felgner J. Smidak R. Choi E. H. Palczewska G. (2025). Safer and efficient base editing and prime editing via ribonucleoproteins delivered through optimized lipid-nanoparticle formulations. Nat. Biomed. Eng. 9, 57–78. 10.1038/s41551-024-01296-2 39609561 PMC11754100

[B15] Hou X. Zaks T. Langer R. Dong Y. (2021). Lipid nanoparticles for mRNA delivery. Nat. Rev. Mater 6, 1078–1094. 10.1038/s41578-021-00358-0 34394960 PMC8353930

[B16] Jinek M. Chylinski K. Fonfara I. Hauer M. Doudna J. A. Charpentier E. (2012). A programmable dual-RNA-guided DNA endonuclease in adaptive bacterial immunity. Science 337, 816–821. 10.1126/science.1225829 22745249 PMC6286148

[B17] Kang S. H. Lee W. J. An J. H. Lee J. H. Kim Y. H. Kim H. (2020). Prediction-based highly sensitive CRISPR off-target validation using target-specific DNA enrichment. Nat. Commun. 11, 3596. 10.1038/s41467-020-17418-8 32681048 PMC7368065

[B18] Kim-Yip R. P. McNulty R. Joyce B. Mollica A. Chen P. J. Ravisankar P. (2024). Efficient prime editing in two-cell mouse embryos using PEmbryo. Nat. Biotechnol. 42, 1822–1830. 10.1038/s41587-023-02106-x 38321114 PMC11631759

[B19] Komor A. C. Kim Y. B. Packer M. S. Zuris J. A. Liu D. R. (2016). Programmable editing of a target base in genomic DNA without double-stranded DNA cleavage. Nature 533, 420–424. 10.1038/nature17946 27096365 PMC4873371

[B20] Lee S. H. Kim S. Hur J. K. (2018). CRISPR and target-specific DNA endonucleases for efficient DNA Knock-in in eukaryotic genomes. Mol. Cells 41, 943–952. 10.14348/molcells.2018.0408 30486613 PMC6277560

[B21] Li T. Yang Y. Qi H. Cui W. Zhang L. Fu X. (2023). CRISPR/Cas9 therapeutics: progress and prospects. Signal Transduct. Target Ther. 8, 36. 10.1038/s41392-023-01309-7 36646687 PMC9841506

[B22] Lino C. A. Harper J. C. Carney J. P. Timlin J. A. (2018). Delivering CRISPR: a review of the challenges and approaches. Drug Deliv. 25, 1234–1257. 10.1080/10717544.2018.1474964 29801422 PMC6058482

[B23] Madigan V. Zhang F. Dahlman J. E. (2023). Drug delivery systems for CRISPR-based genome editors. Nat. Rev. Drug Discov. 22, 875–894. 10.1038/s41573-023-00762-x 37723222

[B24] Oh Y. Gwon L. W. Lee H. K. Hur J. K. Park K. H. Kim K. P. (2024). Highly efficient and specific regulation of gene expression using enhanced CRISPR-Cas12f system. Gene Ther. 31, 358–365. 10.1038/s41434-024-00458-w 38918512

[B25] Pacesa M. Pelea O. Jinek M. (2024). Past, present, and future of CRISPR genome editing technologies. Cell 187, 1076–1100. 10.1016/j.cell.2024.01.042 38428389

[B26] Porto E. M. Komor A. C. Slaymaker I. M. Yeo G. W. (2020). Base editing: advances and therapeutic opportunities. Nat. Rev. Drug Discov. 19, 839–859. 10.1038/s41573-020-0084-6 33077937 PMC7721651

[B27] Rohiwal S. S. Dvorakova N. Klima J. Vaskovicova M. Senigl F. Slouf M. (2020). Polyethylenimine based magnetic nanoparticles mediated non-viral CRISPR/Cas9 system for genome editing. Sci. Rep. 10, 4619. 10.1038/s41598-020-61465-6 32165679 PMC7067791

[B28] She K. Liu Y. Zhao Q. Jin X. Yang Y. Su J. (2023). Dual-AAV split prime editor corrects the mutation and phenotype in mice with inherited retinal degeneration. Signal Transduct. Target Ther. 8, 57. 10.1038/s41392-022-01234-1 36740702 PMC9899767

[B29] Tsuchida C. A. Wasko K. M. Hamilton J. R. Doudna J. A. (2024). Targeted nonviral delivery of genome editors *in vivo* . Proc. Natl. Acad. Sci. U. S. A. 121, e2307796121. 10.1073/pnas.2307796121 38437567 PMC10945750

[B30] Wu F. Li N. Xiao Y. Palanki R. Yamagata H. Mitchell M. J. (2026). Lipid nanoparticles for delivery of CRISPR gene editing components. Small Methods 10, e2401632. 10.1002/smtd.202401632 40434188 PMC12825352

[B31] Xu C. L. Ruan M. Z. C. Mahajan V. B. Tsang S. H. (2019). Viral delivery systems for CRISPR. Viruses 11. 10.3390/v11010028 30621179 PMC6356701

[B32] Yip B. H. (2020). Recent advances in CRISPR/Cas9 delivery strategies. Biomolecules 10. 10.3390/biom10060839 32486234 PMC7356196

[B33] Zhang L. Zuris J. A. Viswanathan R. Edelstein J. N. Turk R. Thommandru B. (2021). AsCas12a ultra nuclease facilitates the rapid generation of therapeutic cell medicines. Nat. Commun. 12, 3908. 10.1038/s41467-021-24017-8 34162850 PMC8222333

[B34] Zhao Z. Shang P. Mohanraju P. Geijsen N. (2023). Prime editing: advances and therapeutic applications. Trends Biotechnol. 41, 1000–1012. 10.1016/j.tibtech.2023.03.004 37002157

