# Supplementary material for: Highly efficient CRISPR editing enabled by magnetic nanoparticle delivery
Source: Front Genome Ed. 2026 Jul 27;8:1865675. doi: 10.3389/fgeed.2026.1865675 (PMC13454498; doi:10.3389/fgeed.2026.1865675)
Supplement: Supplementary file 1 [file DataSheet1.pdf]

Highly efficient CRISPR editing enabled by magnetic nanoparticle delivery

Inkyung Go, Seung Hwan Lee

Supplemental Tables

Supplemental Table S1. SpCas9 target sequence information for gene editing of each target gene. The sequence information encompasses specific target details, comprising both the target sequences and their associated PAM (NGG) sequences. PAM sequences for SpCas9 in the target DNA are shown in yellow.

Gene	Target(protospacer) sequence with PAM (5' to 3')
AAVS1	CTTAGGATGGCCTTCTCCGA**CGG**
DNMT1	CCCTTCAGCTAAAATAAAGG**AGG**
EMX1	AAGCAGCACTCTGCCCTCGT**GGG**
FANCF	GGGGTCCCAGGTGCTGACGT**AGG**
HBB	GCAACCTCAAACAGACACCA**TGG**
RUNX1	ATGAAGCACTGTGGGTACGA**AGG**
HEK4	TCCCTTCCTTCCACCCAGCC**CGG**
PRNP	GCATGTTTTCACGATAGTAA**CGG**
POR	AGCCACGCTCCCTCCACTCA**CGG**
HEXA	CTGGAACTGGTCACCAAGGC**CGG**
CFTR_T1	AGTTTCTTACCTCTTCTAGT**TGG**
CFTR_T2	TATAATTTGGGTAGTGTGAA**GGG**
CFTR_T3	GTAGACTAACCGATTGAATA**TGG**
CFTR_T4	TCACAGTAGCTTACCCATAG**AGG**

Supplemental Table S2. AsCas12a Ultra target sequence information for gene editing of each target gene. The sequence information encompasses specific target details, comprising both the target sequences and their associated PAM (TTTN) sequences. PAM sequences for AsCas12a Ultra in the target DNA are shown in yellow.

Gene	Target(protospacer) sequence with PAM (5' to 3')
AAVS1	**TTTC**TTAGGATGGCCTTCTCCGACGGAT
DNMT1	**TTTC**CTGATGGTCCATGTCTGTTACTCG

EMX1	**TTTG**TCCTCCGGTTCTGGAACCACACCT
FANCF	**TTTG**GTCGGCATGGCCCCATTCGCACGG
HBB	**TTTA**TGGTTGGGATAAGGCTGGATTATT
RUNX1	**TTTC**AGGAGGAAGCGATGGCTTCAGACA
HEK4	**TTTC**CAGTGGTTCAATGGTCATCCCAGG
PRNP	**TTTG**GCTTACTCGGCTTGTTCCACTGAC
POR	**TTTG**GGGTGAGGTCCCAGGTACATACGA
HEXA	**TTTC**ACCTTCAAATGCCAGGGGTTCCAC

Supplemental Table S3. SpCas9(H840A)-RT target sequence information for prime editing of each target gene. The sequence information includes specific target details, comprising both the target sequences and their corresponding PAM (NGG) sequences. PAM sequences for SpCas9(H840A)-RT in the target DNA are shown in yellow.

Gene	Target(protospacer) sequence with PAM (5' to 3')
FANCF	GGAATCCCTTCTGCAGCACC**TGG**
RUNX1	GCATTTTCAGGAGGAAGCGA**TGG**
HEXA	ATCCTTCCAGTCAGGGCCAT**AGG**
EMX1	TGAAGGTGTGGTTCCAGAAC**CGG**
POR	TACCTGGGACCTCACCCCAA**AGG**

Supplemental Table S4. Sequence information for DNA primers used in this study. Primer sequence information to amplify the target gene was indicated. The sequence of the forward and reverse adapter primers used in NGS process is marked in green and blue, respectively.

Target gene (Primer direction)	DNA (5' to 3')
AAVS1_F	CTAAGGATGGGGCTTTTCTGTC
AAVS1_R	TTCTCTGACCTGCATTCTCTCC
DNMT1_F	CACACAGCAGGCCTTTG
DNMT1_R	CCACTCATACAGTGGTAGATTTG
EMX1_F	GGGACACTGGGGATCACTAA
EMX1_R	GAGGAGCTAGGATGCACAG
FANCF_F	AGTTGCTGCACCAGGTGGTA
FANCF_R	CACAGTATGTCTCTGGCGTTAC
HBB_F1	AGGAAGAGATCCATCTACATATCCC

HBB_R1	AGGCCATCACTAAAGGCAC
HBB_F2	CACAGTCTGCCTAGTACATTACTATTTGG
HBB_R2	GGCAGAATCCAGATGCTCAA
HEXA_F	TGCAGGGAGGCCTCTTA
HEXA_R	CTCTCTCTCAGGCCTGAAA
HEK4_F	CTATAGGGGCTTACAGGCGATA
HEK4_R	CCGCAGCCACTCAGAT
POR_F	ACAGGTCGTGGACACTCACA
POR_R	GTCGCAGCAAGGTTAGAAGACA
PRNP_F	CTCTTTGTGGCCACATGG
PRNP_R	ACCACTAAAAGGGCTGCAG
RUNX1_F	ACCACAAGTTGGGTAGCC
RUNX1_R	CACACAGCATGCCGAGTTAA
CFTR_F	CCATTGAGGACGTTTGTCTC
CFTR_R	AGCACTTGGCAACTGTTAGC
EMX1_OT1_F1	CTGGAGATCTAAGAGCCAGT
EMX1_OT1_R1	CCCTTTCTCTCCCACAATTATG
EMX1_OT2_F1	CAGCACCCTCCCTTAACAAA
EMX1_OT2_R1	CAAGGGGATGCAGAGGG
EMX1_OT3_F1	TGTTGAGGTGGGATCGGT
EMX1_OT3_R1	TGCACTTGGCAGAGTGAG
EMX1_OT4_F1	TCCAGCTTTGTTCTTTTGGC
EMX1_OT4_R1	GGGACACTCAGGCAAAAATG
EMX1_OT5_F1	AACTGCATCTGAAAGCTGC
EMX1_OT5_R1	GCTGGGCTCCCAGGAAA
EMX1_OT6_F1	GGAGACTGGATACAAGTCTCTTATT
EMX1_OT6_R1	CTTGGGGTGGGAGGAGT
EMX1_OT7_F1	ATAGGCTGTTTCTGTGTCTCTG
EMX1_OT7_R1	TTACCAGCTACCCCCGTTA
EMX1_OT8_F1	CGAACCAGCCATTGTTAACG
EMX1_OT8_R1	TAGCGCGGAAGCCAGTGAAA
POR_OT1_F1	GAGATAACTGAGGCATTTGGGT
POR_OT1_R1	CTCTGTCTCCCGGGTTCAT
POR_OT2_F1	GTTTCTGTCTACGGCCCAG
POR_OT2_R1	CCTGGTGGGTTTTGCCAG
POR_OT3_F1	ACACTCCAGCTTGTCTGCC
POR_OT3_R1	GCACAAGTCATCATCCTGATGT
POR_OT4_F1	GTACCTGCACTGTAGTTGTGT
POR_OT4_R1	TTGAAGGACATGGCCCTAGG
EMX1_OT1_F2	CAACAGGGGTTATCAGACACTC
EMX1_OT1_R2	CTTAGCCTCTTTGCATTGGG
EMX1_OT2_F2	GCCACTGCTATCACAGCC

EMX1_OT2_R2	CCTATGCCTCTCCCACG
EMX1_OT3_F2	GAAGAGTCTCCCTTCCAG
EMX1_OT3_R2	GTGGATGCATGTCGAAGC
EMX1_OT4_F2	GCCAGGACAGAGAATGG
EMX1_OT4_R2	GAGTAGCTGGGATTACAGGC
EMX1_OT5_F2	GCATGATGCCAGCATTTGC
EMX1_OT5_R2	GCCAGTGTTTGGTGACAG
EMX1_OT6_F2	CATGGAGGATAGCCTAGGG
EMX1_OT6_R2	GTCGTAGCACAGCACGTAC
EMX1_OT7_F2	GACTGCCATTGAGTGAGGC
EMX1_OT7_R2	CACTGCTATATTCCCAGCAGG
EMX1_OT8_F2	CCTGTGACCTCACATAAGCC
EMX1_OT8_R2	CCCAACCAATATGGCTTCTG
EMX1_OT9_F2	GATCAGGAGGTCACGAGTTC
EMX1_OT9_R2	CACATCTGGGCAACAGAAG
EMX1_OT10_F2	CCTTGAAAACTGAGTTCCCAG
EMX1_OT10_R2	GGCTGGCCAATTCCATTAAC
EMX1_OT11_F2	CAGTCACTCATGTCCCTCTG
EMX1_OT11_R2	GGGTTTCACCATGTTGGC
POR_OT1_F2	GTAAACCCCGCAGGAATGT
POR_OT1_R2	CCATCTCTGTCTCAGCCAC
POR_OT2_F2	GTACCTGAACATGGCTGAAAAC
POR_OT2_R2	GTCTCAGATGGGTGTGAAC
POR_OT3_F2	GGTTACCCAGACTGGAC
POR_OT3_R2	CTGACTCAGGTTTGTCAGTTG
POR_OT4_F2	GAGCAGGTGATGAAGCCAAG
POR_OT4_R2	CCATTCATCCCTTCCACAAG
POR_OT5_F2	GCAGTGTAGGGCACTACAG
POR_OT5_R2	GGTACCCTCATTCTGATACACTG
POR_OT6_F2	GTAGATAGATGGGTGGGTGG
POR_OT6_R2	TGCCCAGAGGTTCTGAG
POR_OT7_F2	CCACCCACCGTAATAGG
POR_OT7_R2	CCGTATCGCCTCTCCATTG
POR_OT8_F2	GTGGATCATGAGGTCAGG
POR_OT8_R2	CTGAGGTGGGAGGATTG
POR_OT9_F2	GGATCCTGCTGATTGTGC
POR_OT9_R2	GATTGCAGGCACACACC
POR_OT10_F2	CTCAGCCTCCCAAAGTG
POR_OT10_R2	GGGAATTAGAGAACCAGCC
POR_OT11_F2	GAGAAGGCTCAGAAGCTGAAC
POR_OT11_R2	CAAAAGCTGTCAGGTGGG
POR_OT12_F2	CCCAGCCTTGCAGTAAAAC

POR_OT12_R2	CTTCCGCAGCAATAAAGCG
POR_OT13_F2	CGTGCATTCTTCGCTCTTTG
POR_OT13_R2	CCAGCCTACAATGCTGCTC
POR_OT14_F2	GAACCCATACTGATCCAGCC
POR_OT14_R2	CATAGAGATTCTCCTGGAGCTG
POR_OT15_F2	GTGTGATCCTGGCTCACTG
POR_OT15_R2	GGGTAGAGCAGGTTACTTGTG
AAVS1_adaptor_F	ACACTCTTTCCCTACACGACGCTCTTCCGATCT AAGAGGATGGAGAGGTGG
AAVS1_adaptor_R	GTGACTGGAGTTCAGACGTGTGCTCTTCCGATCT AACCCCATGCCGTCTT
DNMT1_adaptor_F1	ACACTCTTTCCCTACACGACGCTCTTCCGATCT CATCAGTACGTTAATGTTTCCTGATGGT
DNMT1_adaptor_R1	GTGACTGGAGTTCAGACGTGTGCTCTTCCGATCT TCAGTGCATGTTGGGGATTC
DNMT1_adaptor_F2	ACACTCTTTCCCTACACGACGCTCTTCCGATCT AGTGTTCAGTCTCCGTGAAC
DNMT1_adaptor_R2	GTGACTGGAGTTCAGACGTGTGCTCTTCCGATCT TCCTTAGCAGCTTCCTCC
EMX1_adaptor_F1	ACACTCTTTCCCTACACGACGCTCTTCCGATCT GCCATGACTCCAGGCC
EMX1_adaptor_R1	GTGACTGGAGTTCAGACGTGTGCTCTTCCGATCT TCCCATCACATCAACCGG
EMX1_adaptor_F2	ACACTCTTTCCCTACACGACGCTCTTCCGATCT CCGGTTGATGTGATGGG
EMX1_adaptor_R2	GTGACTGGAGTTCAGACGTGTGCTCTTCCGATCT ATCAGGCTCTCAGCTCAG
EMX1_adaptor_F3	ACACTCTTTCCCTACACGACGCTCTTCCGATCT GGTTGCCCACCCTAGTCATTGGAG
EMX1_adaptor_R3	GTGACTGGAGTTCAGACGTGTGCTCTTCCGATCT GCCTCCTGAGTTTCTCATCTGTGC
FANCF_adaptor_F1	ACACTCTTTCCCTACACGACGCTCTTCCGATCT ACCAAAGCGCCGATGGAT
FANCF_adaptor_R1	GTGACTGGAGTTCAGACGTGTGCTCTTCCGATCT CAGAGAGTCGCCGTCTC
FANCF_adaptor_F2	ACACTCTTTCCCTACACGACGCTCTTCCGATCT ACGTCACAGTGACCGAG
FANCF_adaptor_R2	GTGACTGGAGTTCAGACGTGTGCTCTTCCGATCT TACGTCAGCACCTGGG
FANCF_adaptor_R3	GTGACTGGAGTTCAGACGTGTGCTCTTCCGATCT TCGCGGATGTTCCAATCAGTA
HBB_adaptor_F1	ACACTCTTTCCCTACACGACGCTCTTCCGATCT CTCCTTAAACCTGTCTTGTAACCTTG

HBB_adaptor_R1	GTGACTGGAGTTCAGACGTGTGCTCTTCCGATCT GTCAGGGCAGAGCCAT
HBB_adaptor_F2	ACACTCTTTCCCTACACGACGCTCTTCCGATCT GTAACTGATGTAAGAGGTTTCATATTGCTAATAGCAG
HBB_adaptor_R2	GTGACTGGAGTTCAGACGTGTGCTCTTCCGATCT CTTTGCCAAAGTGATGGGC
HEXA_adaptor_F1	ACACTCTTTCCCTACACGACGCTCTTCCGATCT CAGCTTTGTGTCCTTACTGCC
HEXA_adaptor_R1	GTGACTGGAGTTCAGACGTGTGCTCTTCCGATCT GTAGAAATCCTTCCAGTCAGGG
HEXA_adaptor_F2	ACACTCTTTCCCTACACGACGCTCTTCCGATCT GAACTGGTCACCAAGGC
HEXA_adaptor_R2	GTGACTGGAGTTCAGACGTGTGCTCTTCCGATCT GTCTGGCCCAGACCTT
HEXA_adaptor_F3	ACACTCTTTCCCTACACGACGCTCTTCCGATCT TGTGGCGAGAGGATATTCCA
HEXA_adaptor_R3	GTGACTGGAGTTCAGACGTGTGCTCTTCCGATCT CCTTTGGTTAGCAAGGAGAG
HEK4_adaptor_F1	ACACTCTTTCCCTACACGACGCTCTTCCGATCT ACCCAGGTAGCCAGAGA
HEK4_adaptor_R1	GTGACTGGAGTTCAGACGTGTGCTCTTCCGATCT CAGAGTCTCCGCTTTAACCC
HEK4_adaptor_F2	ACACTCTTTCCCTACACGACGCTCTTCCGATCT CGTGGAGACAGACCACAA
HEK4_adaptor_R2	GTGACTGGAGTTCAGACGTGTGCTCTTCCGATCT AGAAGACCAGCGGGTC
POR_adaptor_F1	ACACTCTTTCCCTACACGACGCTCTTCCGATCT GTGAGAAGGGCCCTGT
POR_adaptor_R1	GTGACTGGAGTTCAGACGTGTGCTCTTCCGATCT TCGCCAGTACGAGCTTGT
POR_adaptor_F2	ACACTCTTTCCCTACACGACGCTCTTCCGATCT GAAAGGGGAGACCAAGG
POR_adaptor_R2	GTGACTGGAGTTCAGACGTGTGCTCTTCCGATCT TTCTGGGCGTCTGGC
POR_adaptor_F3	ACACTCTTTCCCTACACGACGCTCTTCCGATCT GCTGGAAAGGGGAGACCAAGGG
POR_adaptor_R3	GTGACTGGAGTTCAGACGTGTGCTCTTCCGATCT TCCACGCCGCCTCCCTCC
PRNP_adaptor_F1	ACACTCTTTCCCTACACGACGCTCTTCCGATCT TGCTGCAGCAGCTGG
PRNP_adaptor_R1	GTGACTGGAGTTCAGACGTGTGCTCTTCCGATCT GATATTGACGCAGTCGTGCAC
PRNP_adaptor_F2	ACACTCTTTCCCTACACGACGCTCTTCCGATCT

	TACCCACCTCAGGGC
PRNP_adaptor_R2	GTGACTGGAGTTCAGACGTGTGCTCTTCCGATCT CTGCAGCACCAGCCAT
RUNX1_adaptor_F1	ACACTCTTTCCCTACACGACGCTCTTCCGATCT CGAAGGTAAAAGAAATCATTGAGTCCC
RUNX1_adaptor_R1	GTGACTGGAGTTCAGACGTGTGCTCTTCCGATCT CCGAGGTGAAACAAGCTGC
RUNX1_adaptor_F2	ACACTCTTTCCCTACACGACGCTCTTCCGATCT AACAGTGACCTGTCTTGGTTTTC
RUNX1_adaptor_R2	GTGACTGGAGTTCAGACGTGTGCTCTTCCGATCT TACAGGCAAAGCTGAGCAAAAGT
RUNX1_adaptor_F3	ACACTCTTTCCCTACACGACGCTCTTCCGATCT AGGGGTGAGGCTGAAACA
RUNX1_adaptor_R3	GTGACTGGAGTTCAGACGTGTGCTCTTCCGATCT GCTGCCATTTCATTACAGGC
CFTR_adaptor_F1	ACACTCTTTCCCTACACGACGCTCTTCCGATCT GTGTGAAGGGTTCATATGCATAATC
CFTR_adaptor_R1	GTGACTGGAGTTCAGACGTGTGCTCTTCCGATCT GGAGCCTTCAGAGGGTAAAATT
CFTR_adaptor_F2	ACACTCTTTCCCTACACGACGCTCTTCCGATCT CATGTGTTTGCAAGCTTCTTAAAGCATAGG
CFTR_adaptor_R2	GTGACTGGAGTTCAGACGTGTGCTCTTCCGATCT GCATGCCAACTAGAAGAGGTAAG
EMX1_OT1_adaptor_F1	ACACTCTTTCCCTACACGACGCTCTTCCGATCT TTGTAAACTGTGTCTGAGGG
EMX1_OT1_adaptor_R1	GTGACTGGAGTTCAGACGTGTGCTCTTCCGATCT GCATAGCCTGTGGGTCTG
EMX1_OT2_adaptor_F1	ACACTCTTTCCCTACACGACGCTCTTCCGATCT ACTGTCCTCGTCTCTCCTTCT
EMX1_OT2_adaptor_R1	GTGACTGGAGTTCAGACGTGTGCTCTTCCGATCT GGTGGCCGTGATGTCGT
EMX1_OT3_adaptor_F1	ACACTCTTTCCCTACACGACGCTCTTCCGATCT CTGGCACCGCGATCCT
EMX1_OT3_adaptor_R1	GTGACTGGAGTTCAGACGTGTGCTCTTCCGATCT GAGCGGCGCTTCAAGCA
EMX1_OT4_adaptor_F1	ACACTCTTTCCCTACACGACGCTCTTCCGATCT TTGGAATGCTTTCAGCTACCA
EMX1_OT4_adaptor_R1	GTGACTGGAGTTCAGACGTGTGCTCTTCCGATCT TTGCAACTGCAGAAAGCTTC
EMX1_OT5_adaptor_F1	ACACTCTTTCCCTACACGACGCTCTTCCGATCT CCTCCTCTACTTTCTCGGAG
EMX1_OT5_adaptor_R1	GTGACTGGAGTTCAGACGTGTGCTCTTCCGATCT CACAGCTTCTAGAGCTGGAA

EMX1_OT6_adaptor_F1	ACACTCTTTCCCTACACGACGCTCTTCCGATCT TGGAGACAGGCTGGAAAGA
EMX1_OT6_adaptor_R1	GTGACTGGAGTTCAGACGTGTGCTCTTCCGATCT ATGTGCCAAGAGCATCCC
EMX1_OT7_adaptor_F1	ACACTCTTTCCCTACACGACGCTCTTCCGATCT GCCAGGCGGTGAAGG
EMX1_OT7_adaptor_R1	GTGACTGGAGTTCAGACGTGTGCTCTTCCGATCT TCCAGGCGGTCCACCA
EMX1_OT8_adaptor_F1	ACACTCTTTCCCTACACGACGCTCTTCCGATCT CCTGCTCCAGCAGCC
EMX1_OT8_adaptor_R1	GTGACTGGAGTTCAGACGTGTGCTCTTCCGATCT GGGAGCTCGCTTCCTTT
POR_OT1_adaptor_F1	ACACTCTTTCCCTACACGACGCTCTTCCGATCT GGGGGCTATTATCATCCCAT
POR_OT1_adaptor_R1	GTGACTGGAGTTCAGACGTGTGCTCTTCCGATCT GGGTGTACACACCCATACAC
POR_OT2_adaptor_F1	ACACTCTTTCCCTACACGACGCTCTTCCGATCT GGCAGAAGAGGCCCTCAG
POR_OT2_adaptor_R1	GTGACTGGAGTTCAGACGTGTGCTCTTCCGATCT AGAAGGCAGACTAAAAGTAGGC
POR_OT3_adaptor_F1	ACACTCTTTCCCTACACGACGCTCTTCCGATCT ATCCCAGCTAGAGCCTGG
POR_OT3_adaptor_R1	GTGACTGGAGTTCAGACGTGTGCTCTTCCGATCT GGTCCAGCCTGGACACC
POR_OT4_adaptor_F1	ACACTCTTTCCCTACACGACGCTCTTCCGATCT CAAAGGGCTAAGTAACTTCTCC
POR_OT4_adaptor_R1	GTGACTGGAGTTCAGACGTGTGCTCTTCCGATCT CATGAGTGAATGGGCACAC
EMX1_OT1_adaptor_F2	ACACTCTTTCCCTACACGACGCTCTTCCGATCT GGAGTATGTTAGTAGCCTCAAAG
EMX1_OT1_adaptor_R2	GTGACTGGAGTTCAGACGTGTGCTCTTCCGATCT GAGCTAAAGAACCACCTTCTG
EMX1_OT2_adaptor_F2	ACACTCTTTCCCTACACGACGCTCTTCCGATCT GCAAAATCTAGAGATGGGGC
EMX1_OT2_adaptor_R2	GTGACTGGAGTTCAGACGTGTGCTCTTCCGATCT GAATTTGATCCTGGGCTCC
EMX1_OT3_adaptor_F2	ACACTCTTTCCCTACACGACGCTCTTCCGATCT GCCTTCAGTTAGATGGTCAGAC
EMX1_OT3_adaptor_R2	GTGACTGGAGTTCAGACGTGTGCTCTTCCGATCT CTAGTGTTCTGTGGCTGAGG
EMX1_OT4_adaptor_F2	ACACTCTTTCCCTACACGACGCTCTTCCGATCT GCCCTCTATAGAGTGCCAG
EMX1_OT4_adaptor_R2	GTGACTGGAGTTCAGACGTGTGCTCTTCCGATCT CCCCGGTCTGTAATAATGACTC
EMX1_OT5_adaptor_F2	ACACTCTTTCCCTACACGACGCTCTTCCGATCT GATTTGGCGGGTCCAAAC

EMX1_OT5_adaptor_R2	GTGACTGGAGTTCAGACGTGTGCTCTTCCGATCT GACACTTAGTGATGGCTTGTGTAG
EMX1_OT6_adaptor_F2	ACACTCTTTCCCTACACGACGCTCTTCCGATCT CTCTGCTGCTCCCTTCTG
EMX1_OT6_adaptor_R2	GTGACTGGAGTTCAGACGTGTGCTCTTCCGATCT GACGCCAGGGTCAGG
EMX1_OT7_adaptor_F2	ACACTCTTTCCCTACACGACGCTCTTCCGATCT CTGAGATTGCGCCACTG
EMX1_OT7_adaptor_R2	GTGACTGGAGTTCAGACGTGTGCTCTTCCGATCT CCTATTCTCTATAACCTCAGAGGAG
EMX1_OT8_adaptor_F2	ACACTCTTTCCCTACACGACGCTCTTCCGATCT CTGGCTGCCTGGAAC
EMX1_OT8_adaptor_R2	GTGACTGGAGTTCAGACGTGTGCTCTTCCGATCT GGCCAAAGGTGTTCATGTTC
EMX1_OT9_adaptor_F2	ACACTCTTTCCCTACACGACGCTCTTCCGATCT CCCTTTCTCGTGCAGC
EMX1_OT9_adaptor_R2	GTGACTGGAGTTCAGACGTGTGCTCTTCCGATCT GTTTATTTTAGGGGGACATGGTG
EMX1_OT10_adaptor_F2	ACACTCTTTCCCTACACGACGCTCTTCCGATCT GGGCTAACCAGAAACTAGCC
EMX1_OT10_adaptor_R2	GTGACTGGAGTTCAGACGTGTGCTCTTCCGATCT GCTGAAGTCTGGAGCC
EMX1_OT11_adaptor_F2	ACACTCTTTCCCTACACGACGCTCTTCCGATCT CCACATGTCACTGAATGCCTAC
EMX1_OT11_adaptor_R2	GTGACTGGAGTTCAGACGTGTGCTCTTCCGATCT CTTCCTTTTAAGTATGCTAGATTTGCTC
POR_OT1_adaptor_F2	ACACTCTTTCCCTACACGACGCTCTTCCGATCT GCCCATCGTATGTCTTCTTTACAG
POR_OT1_adaptor_R2	GTGACTGGAGTTCAGACGTGTGCTCTTCCGATCT CCTTTTAACAGAGAACCATCAAGACC
POR_OT2_adaptor_F2	ACACTCTTTCCCTACACGACGCTCTTCCGATCT CAAGCACTGCACGCAC
POR_OT2_adaptor_R2	GTGACTGGAGTTCAGACGTGTGCTCTTCCGATCT CATTTGACCCACGCAGTG
POR_OT3_adaptor_F2	ACACTCTTTCCCTACACGACGCTCTTCCGATCT GTGTTAGAGACGTGCATTCCTC
POR_OT3_adaptor_R2	GTGACTGGAGTTCAGACGTGTGCTCTTCCGATCT CTGGGAGGAACTTGAACG
POR_OT4_adaptor_F2	ACACTCTTTCCCTACACGACGCTCTTCCGATCT CACCCCAGATGGCTTTG
POR_OT4_adaptor_R2	GTGACTGGAGTTCAGACGTGTGCTCTTCCGATCT CAAGTGCTCAGTGGAGGTC
POR_OT5_adaptor_F2	ACACTCTTTCCCTACACGACGCTCTTCCGATCT GGTGTCCTGCAGTCTGATC
POR_OT5_adaptor_R2	GTGACTGGAGTTCAGACGTGTGCTCTTCCGATCT CATGTTGTGCGCTTCCC
POR_OT6_adaptor_F2	ACACTCTTTCCCTACACGACGCTCTTCCGATCT GAATTAGTGTGCAGCTGGTGG
POR_OT6_adaptor_R2	GTGACTGGAGTTCAGACGTGTGCTCTTCCGATCT GTTCTCTCCTACAAAATCAAGTGCTC
POR_OT7_adaptor_F2	ACACTCTTTCCCTACACGACGCTCTTCCGATCT

	CACTTGTACATGACTACACTGAGC
POR_OT7_adaptor_R2	GTGACTGGAGTTCAGACGTGTGCTCTTCCGATCT GATTGCGCCACTGCAATC
POR_OT8_adaptor_F2	ACACTCTTTCCCTACACGACGCTCTTCCGATCT GAGGGTCCTTCACAAGAC
POR_OT8_adaptor_R2	GTGACTGGAGTTCAGACGTGTGCTCTTCCGATCT GCTATTCCACATACAACATTCCAGC
POR_OT9_adaptor_F2	ACACTCTTTCCCTACACGACGCTCTTCCGATCT GTCAGGTAAGGCCTGATAAGG
POR_OT9_adaptor_R2	GTGACTGGAGTTCAGACGTGTGCTCTTCCGATCT CAGCCTCCTGCTTAAGTGC
POR_OT10_adaptor_F2	ACACTCTTTCCCTACACGACGCTCTTCCGATCT GGGCAAGGGGTTTGAAG
POR_OT10_adaptor_R2	GTGACTGGAGTTCAGACGTGTGCTCTTCCGATCT CAATCTGCCCATTTTTGTACCTTC
POR_OT11_adaptor_F2	ACACTCTTTCCCTACACGACGCTCTTCCGATCT CTCTTGCGACCGGTGAC
POR_OT11_adaptor_R2	GTGACTGGAGTTCAGACGTGTGCTCTTCCGATCT TGTCTGGGAGGAGGG
POR_OT12_adaptor_F2	ACACTCTTTCCCTACACGACGCTCTTCCGATCT GTCGGGAAGCTTGCTTG
POR_OT12_adaptor_R2	GTGACTGGAGTTCAGACGTGTGCTCTTCCGATCT GAAAGCATCGGTGTTTGCG
POR_OT13_adaptor_F2	ACACTCTTTCCCTACACGACGCTCTTCCGATCT GAGAGCCTTGGTGTGAGAAG
POR_OT13_adaptor_R2	GTGACTGGAGTTCAGACGTGTGCTCTTCCGATCT GACACAGGCGGAAGAAAAAC
POR_OT14_adaptor_F2	ACACTCTTTCCCTACACGACGCTCTTCCGATCT CCTACCATTACTCACCGTTTCC
POR_OT14_adaptor_R2	GTGACTGGAGTTCAGACGTGTGCTCTTCCGATCT CTTCTCCCTGGAACCTG
POR_OT15_adaptor_F2	ACACTCTTTCCCTACACGACGCTCTTCCGATCT CTTTAGTAGGCATTCCCAGGG
POR_OT15_adaptor_R2	GTGACTGGAGTTCAGACGTGTGCTCTTCCGATCT GTATACAGGCACTGGGGC

Supplemental Table S5. Sequence information of in-silico base predicted off-target sites for each target sites measured in this study. Target and off-target sequences and position on the chromosome are indicated, respectively. Mismatch sequences are shown in red. PAM sequences (NGG) in the target DNA for SpCas9(H840A)-RT are highlighted in yellow.

Gene		DNA	Chromosome	Position	Mismatches
POR	On	TACCTGGGACCTCACCCCAA**AGG**	chr7	75982110	0
	OT1	gACCTGGGACCTgAgCCCAA**AGG**	chr12	3966972	3
	OT2	TcCCTGGGACCgCcCCCCAA**AGG**	chr4	2251908	3
	OT3	gcCCTGGGACCcCACCCCAA**AGG**	chr16	88688422	3

	OT4	TACCTGGaACCTCACagCAA**AGG**	chr6	103844021	3
EMX1	On	TGAAGGTGTGGTTCCAGAAC**CGG**	chr2	72933787	0
	OT1	aGAAtGTGTGtTTCCAGAAC**TGG**	chr5	7412664	3
	OT2	TGAAGaTcTGGTTCCAaAAC**CGG**	chr5	150183474	3
	OT3	TcAAGaTcTGGTTCCAGAAC**CGG**	chr5	173232976	3
	OT4	TGAttGgGTGGTTCCAGAAC**TGG**	chr1	103288143	3
	OT5	TGAAaGTGTGGTTtCAGAAC**CGG**	chr7	27101274	2
	OT6	TGcAGGTGTGGTTCaAGAAC**CGG**	chr2	127025017	2
	OT7	TGcAGGTcTGGTTCCAGAAC**CGG**	chr17	36942194	2
	OT8	TGAAGGTcTGGTTCCAGAAC**CGG**	chr10	117134559	1

Supplemental Figures

AAVS1

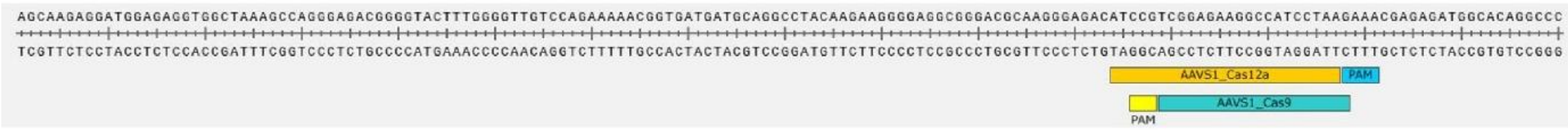

DNMT1

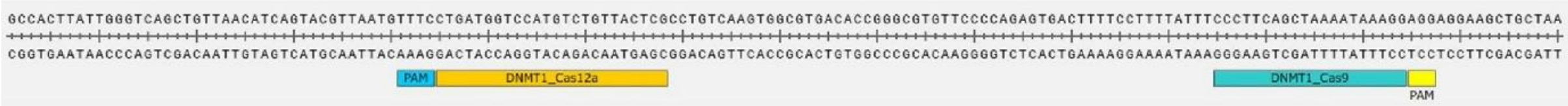

EMX1

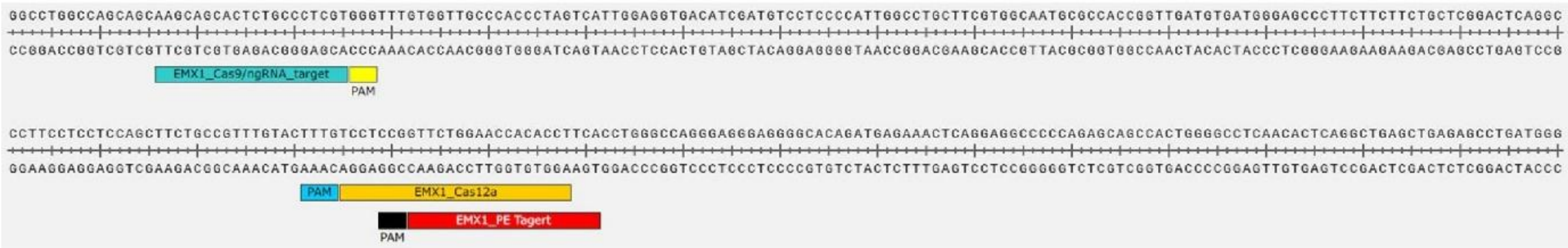

FANCF

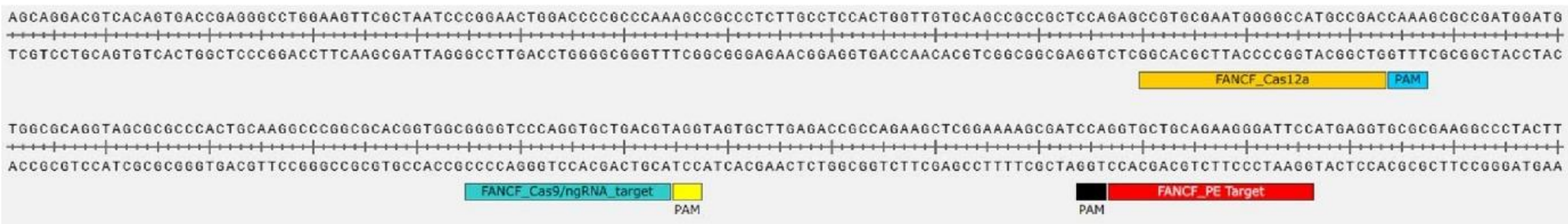

HBB

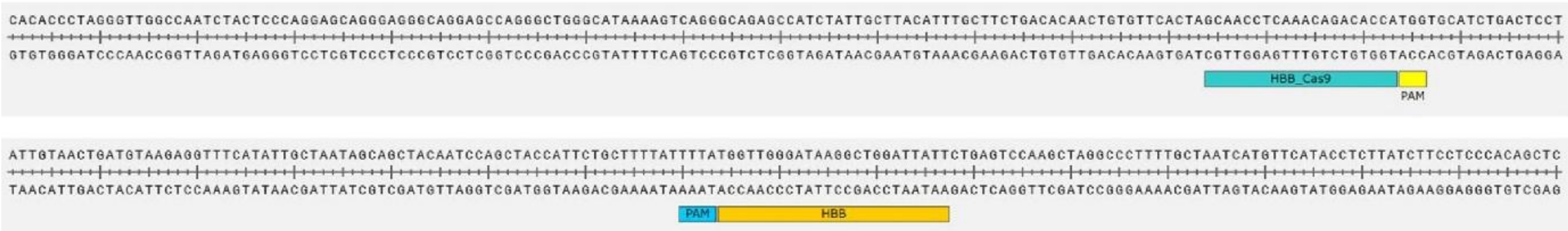

RUNX1

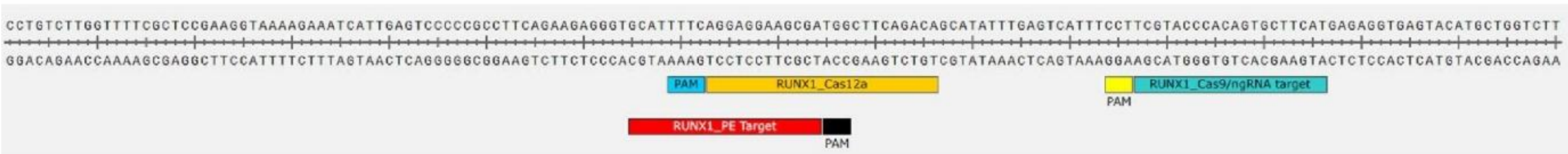

HEK4

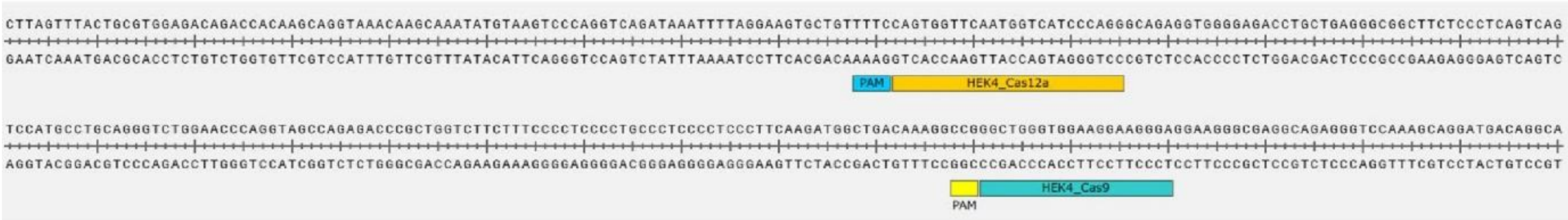

PRNP

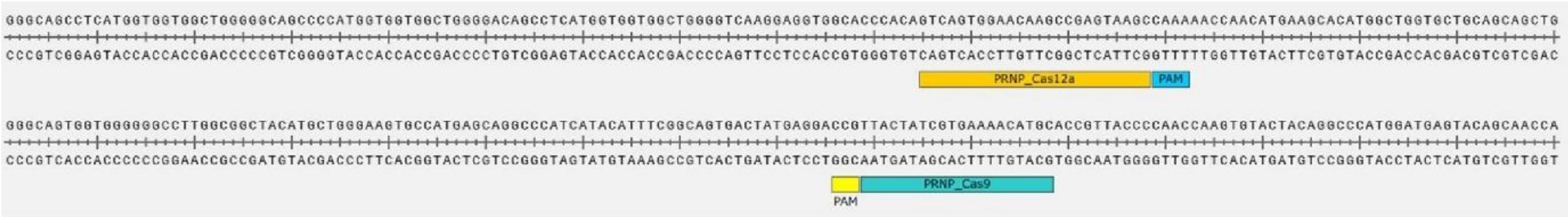

POR

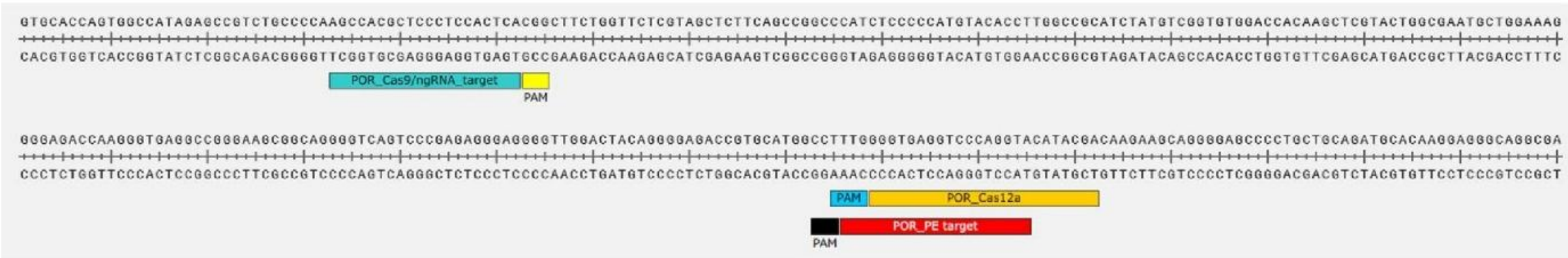

HEXA

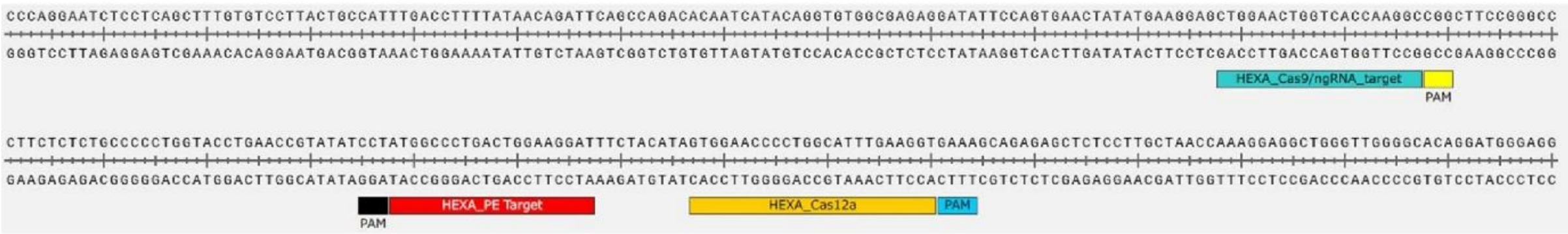

CuFi-1(CFTR)

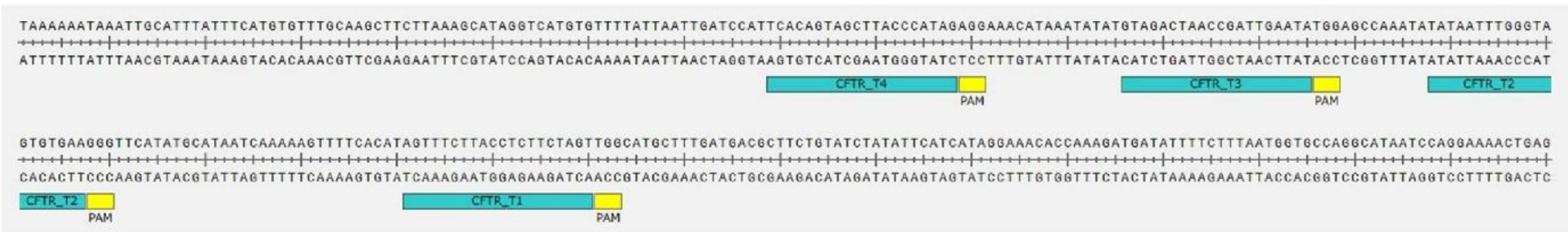

Supplemental Figure S1. Schematic representation of guide RNA target sites used for SpCas9, AsCas12a, and prime editing. Genomic sequence maps showing the target regions used for CRISPR-mediated genome editing at *the AAVS1, DNMT1, EMX1, FANCF, HBB, RUNX1, HEK4, PRNP, POR, HEXA*, and *CFTR* loci. Cyan bars indicate SpCas9 sgRNA target sites, orange bars indicate AsCas12a crRNA target sites, and red bars indicate prime editing target sites, respectively. The nicking guide RNA (ngRNA) target regions used for prime editing are also indicated (Cyan bars) where applicable. PAM positions (yellow for Cas9, blue for Cas12a, and black for prime editor, respectively) are shown adjacent to each target sequence. The *AAVS1* target site used for RNP delivery was identical to the SpCas9 sgRNA target site shown in this schematic. These schematics summarize the guide RNA target designs used for the SpCas9-, AsCas12a-, RNP-, and prime editing experiments performed under LIGE

and MAGE delivery conditions. LIGE: lipofection-mediated genome editing, MAGE: magnetofection-mediated genome editing.

A

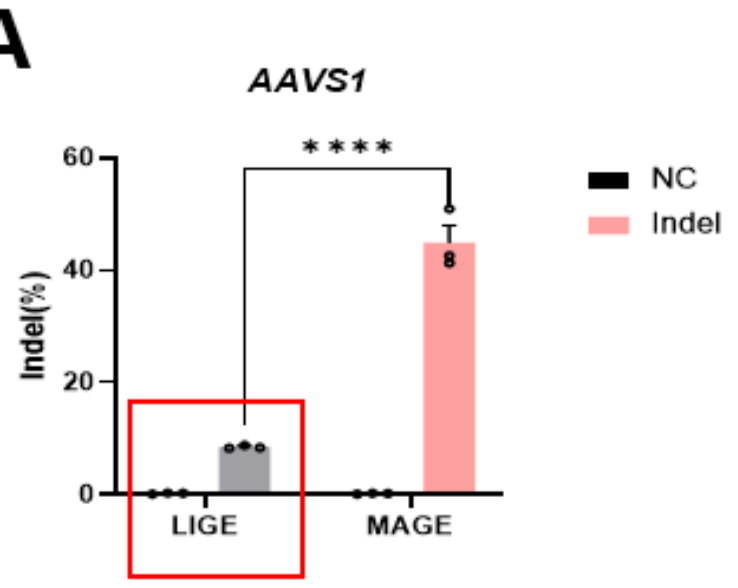

B **SpCas9_Lipofection(LIGE)**

CTTAGGATGGCCTTCTCCGACGGATGTCTCCC (wt)
CTTAGGATGGCCTTCT –CGACGGATGTCTCCC (1bp del)
CTTAGGATGGCCT - - - - - - - - - - - - - TCTCCC (13bp del)
CTTA - - - - - - - - - - - - - - - - - GGATGTCTCCC (17bp del)
CTTAGGATGGCCTTCTCCCGACGGATGTCTCCC (1bp ins)
CTTAGGATGGCCTTCTC - - - - - - - - GTCTCCC (8bp del)
CTTAGGATGGCCTTCTC - - - - - - ATGTCTCCC (6bp del)
CTTAGGATGGCCTTCT - - - - CGGATGTCTCCC (4bp del)
CTTAGGATGGC - - - - - CCGACGGATGTCTCCC (5bp del)

C

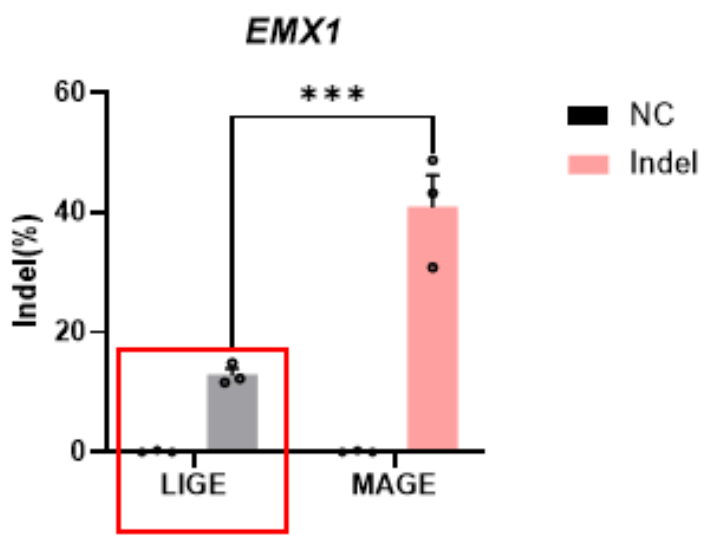

D **SpCas9_Lipofection(LIGE)**

AAGCAGCACTCTGCCCTCGTGGGTTTGTGGTTG (wt)
AAGCAGCACTCTGCCCTTCGTGGGTTTGTGGTTG (1bp ins)
AAGCAGCACTCTGCC - -CGTGGGTTTGTGGTTG (2bp del)
AAGCAG - - - - - - - - - - - CGTGGGTTTGTGGTTG (11bp del)
AAGCAGCACTCTGCCCTCTCGTGGGTTTGTGGTTG (2bp ins)
AAGCAGCACTCTG - - - - CGTGGGTTTGTGGTTG (4bp del)
AAGCAGCACTCTGCCCTACGTGGGTTTGTGGTTG (1bp ins)
AAGCAGCACTC - TG (20bp del)
AAGCAGCACTCTGC - - -CGTGGGTTTGTGGTTG (3bp del)
AAGCAGCA - - - - - - -CTCGTGGGTTTGTGGTTG (7bp del)

E

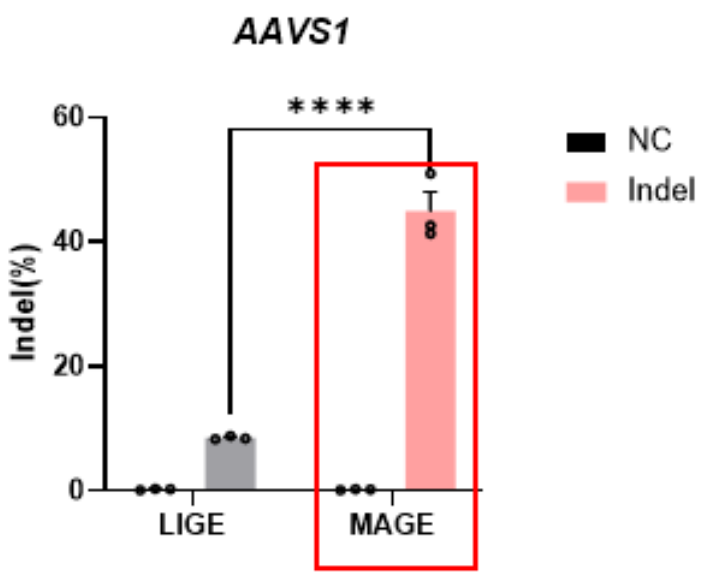

F **SpCas9_Magnetofection(MAGE)**

CTTAGGATGGCCTTCTCCGACGGATGTCTCCC (wt)
CTTAGGATGGCCTTCT -CGACGGATGTCTCCC (1bp del)
CTTA - - - - - - - - - - - - - - - - - GGATGTCTCCC (17bp del)
CTTAGGATGGCCTTCTCCCGACGGATGTCTCCC (1bp ins)
CTTAGGATGGCCTTCTCGCGACGGATGTCTCCC (1bp ins)
CTTAGGATGGCCT - - - - - - - - - - - - - TCTCCC (13bp del)
CTTAGGATGGCCTTCTCACGACGGATGTCTCCC (1bp ins)
CTTAGGATGGCCTTCTC - - - - - - - - GTCTCCC (8bp del)
CTTAGGATGGCCTTCT - - - - CGGATGTCTCCC (4bp del)
CTTAGGATGGCCTTC - - - - - - - - - - - TCTCCC (11bp del)

G

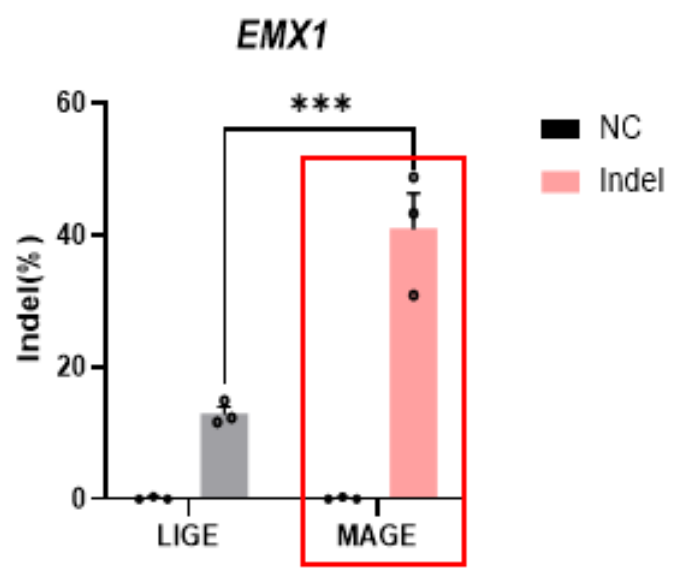

H **SpCas9_Magnetofection(MAGE)**

AAGCAGCACTCTGCCCTCGTGGGTTTGTGGTTG (wt)
AAGCAGCACTCTGCCCTTCGTGGGTTTGTGGTTG (1bp ins)
AAGCAGCACTCTGCCCTCTCGTGGGTTTGTGGTTG (2bp ins)
AAGCAGCACTCTGCC - - CGTGGGTTTGTGGTTG (2bp del)
AAGCAGCACTCTGCCC - CGTGGGTTTGTGGTTG (1bp del)
AAGCAGCACTCTG - - - - CGTGGGTTTGTGGTTG (4bp del)
AAGCAGCACTCTGCCCT - GTGGGTTTGTGGTTG (1bp del)
AAGCAGCACTC - TG (20bp del)
AAGCAGCACT- - - - - - - CGTGGGTTTGTGGTTG(7bp del)
AAGCAGCACTCTGCCCTACGTGGGTTTGTGGTTG(1bp ins)

I

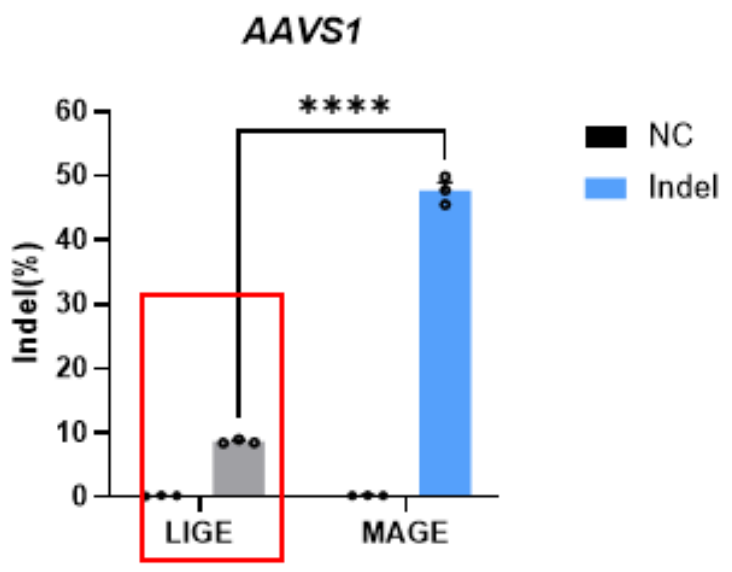

J

AsCas12a_Lipofection(LIGE)

TTTCTTAGGATGGCCTTCTCCGACGGATGTCTCCCTTGCG (wt)
TTTCTTA - - - - - - - - - - - - - - - - - GGATGTCTCCCTTGCG (17bp del)
TTTCTTAGGATGG - CCTTGCG (20bp del)
TTTCTTAGGATGGCCTT - - - - - - CGGATGTCTCCCTTGCG (6bp del)
TTTCTTAGGATGGCCTTCTC - - - CGGATGTCTCCCTTGCG (3bp del)
TTTCTTAGGATGGCCT - - - - - - - - - - - - - TCTCCCTTGCG (13bp del)
GTTTCTTAGGATGGCCTTCT- - - -CGGATGTCTCCCTTGCG (4bp del)
GTTTCTTAGGATGGC - - - - - - - - - - - - - - - - CTCCCTTGCG (16bp del)
TTTCTTAGGATGGCCTT - - - - - - - - - - - - - - - - - CTTGCG (17bp del)
TTTCTTAGGATGGCCTTCTCC - - - - GATGTCTCCCTTGCG (4bp del)

K

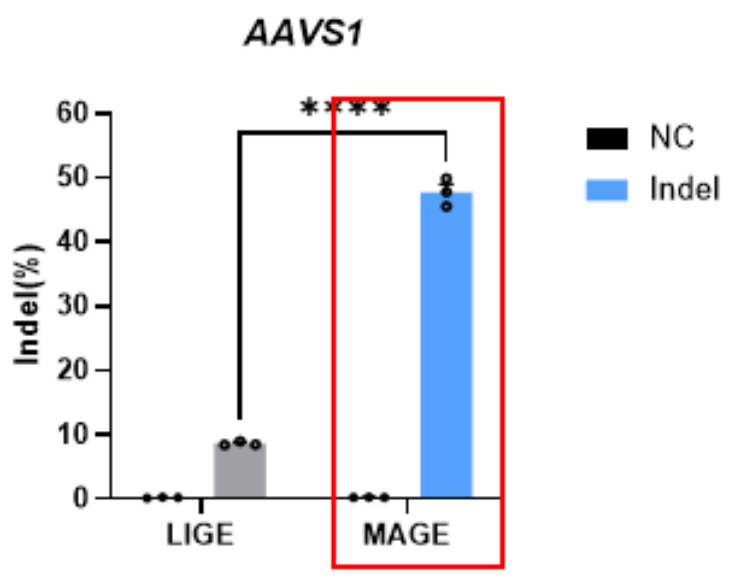

L

AsCas12a_Magnetofection(MAGE)

TTTCTTAGGATGGCCTTCTCCGACGGATGTCTCCCTTGCG (wt)
TTTCTTAGGATGGCCT - - - - - - - - - - - - - TCTCCCTTGCG (13bp del)
TTTCTTAGGATGGCCTT - - - - - - CGGATGTCTCCCTTGCG (6bp del)
TTTCTTA - - - - - - - - - - - - - - - - - GGATGTCTCCCTTGCG (17bp del)
TTTCTTAGGATGG - CCTTGCG (20bp del)
TTTCTTAGGATGGCCTTCTC - - - CGGATGTCTCCCTTGCG (3bp del)
TTTCTTAGGATGGCCTTCT - - - - CGGATGTCTCCCTTGCG (4bp del)
TTTCTTAGGATGGCCT - - - - - - - - - - - TGTCTCCCTTGCG (11bp del)
TTTCTTAGGATGGCCTTC - - - - ACGGATGTCTCCCTTGCG (4bp del)
TTTCTTAGGATGGCCTT - - - - - - - - - - - - - - - - CCTTGCG (16bp del)

M

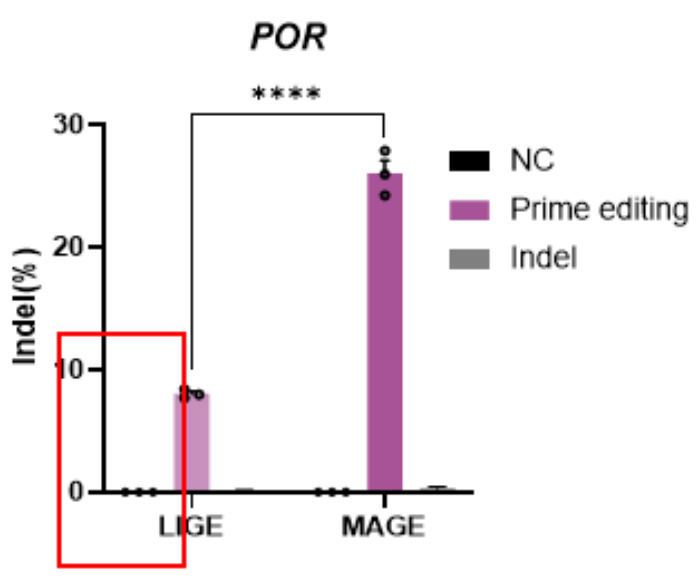

N

Prime editor_Lipofection(LIGE)

TATGTACCTGGGACCTCACCCCAAAGG (wt)
TATGTACCTGGGACCTCACCCTTCAAAGG (TT ins: precise edit)

O

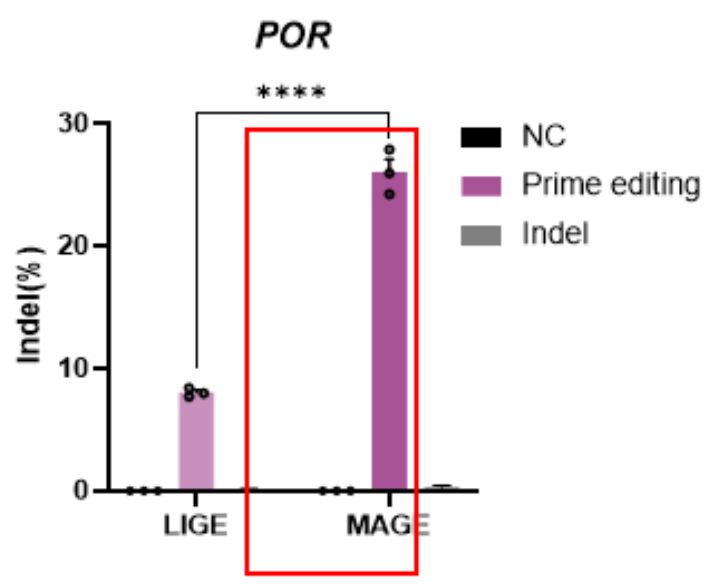

P

Prime editor_Magnetofection(MAGE)

TATGTACCTGGGACCTCACCCCAAAGG (wt)
TATGTACCTGGGACCTCACCCTTCAAAGG (TT ins: precise edit)
TATGTACCTGGGACCTCACCCTTCAAAGGCCAAAGG (9bp ins: unintended edit)
TATGTACCTGGGACCTCACCCTTCAAAGGCCATGCAGCTCAAAGG (18bp ins: unintended edit)
TATGTACCTGGGACCTCACCCTTCAAAGGCCATGCAGCCAAAGG (17bp ins: unintended edit)

Supplemental Figure S2. Representative NGS-based mutation pattern analysis following genome editing using SpCas9, AsCas12a, and prime editor under LIGE and MAGE delivery conditions. Genome editing patterns generated by SpCas9 (A–H), AsCas12a (I–L), and prime editor (M–P) were compared between LIGE (lipofection-mediated genome editing) and MAGE (magnetofection-mediated genome editing) delivery conditions. Each histogram shown here corresponds directly to the histograms presented in the main figures, and the red boxes indicate the samples selected for mutation pattern analysis. Histograms A, C, E, and G correspond

to a subset of data from main Figure 1A, whereas histograms I and K correspond to data from main Figure 1C. Histograms M and O correspond to data from main Figure 3A. Sequencing results shown next to each histogram show representative mutation patterns observed at the edited loci. B, D, F, H: representative mutation patterns obtained following genome editing induced by delivery of the SpCas9 expression vector via lipofection and magnetofection, respectively. J, L: representative mutation patterns generated following genome editing induced by delivery of the AsCas12a expression vector via lipofection and magnetofection, respectively. N, P: representative mutation patterns generated following genome editing induced by delivery of the prime editor expression vectors via lipofection and magnetofection, respectively. PAM and target sequences are shown in red and yellow, respectively. Inserted sequences are highlighted in blue and deleted sequences are shown in dashed line. Data are presented as mean ± standard error from three independent biological replicates. Statistical significance was evaluated using two-way ANOVA (ns: not significant, *$p \leq 0.05$, **$p \leq 0.01$, ***$p \leq 0.001$, ****$p \leq 0.0001$). NC: negative control, Indel: insertion and deletion patterns, Prime editing: precise mutation introduced by prime editing. LIGE: lipofection-mediated genome editing, MAGE: magnetofection-mediated genome editing.

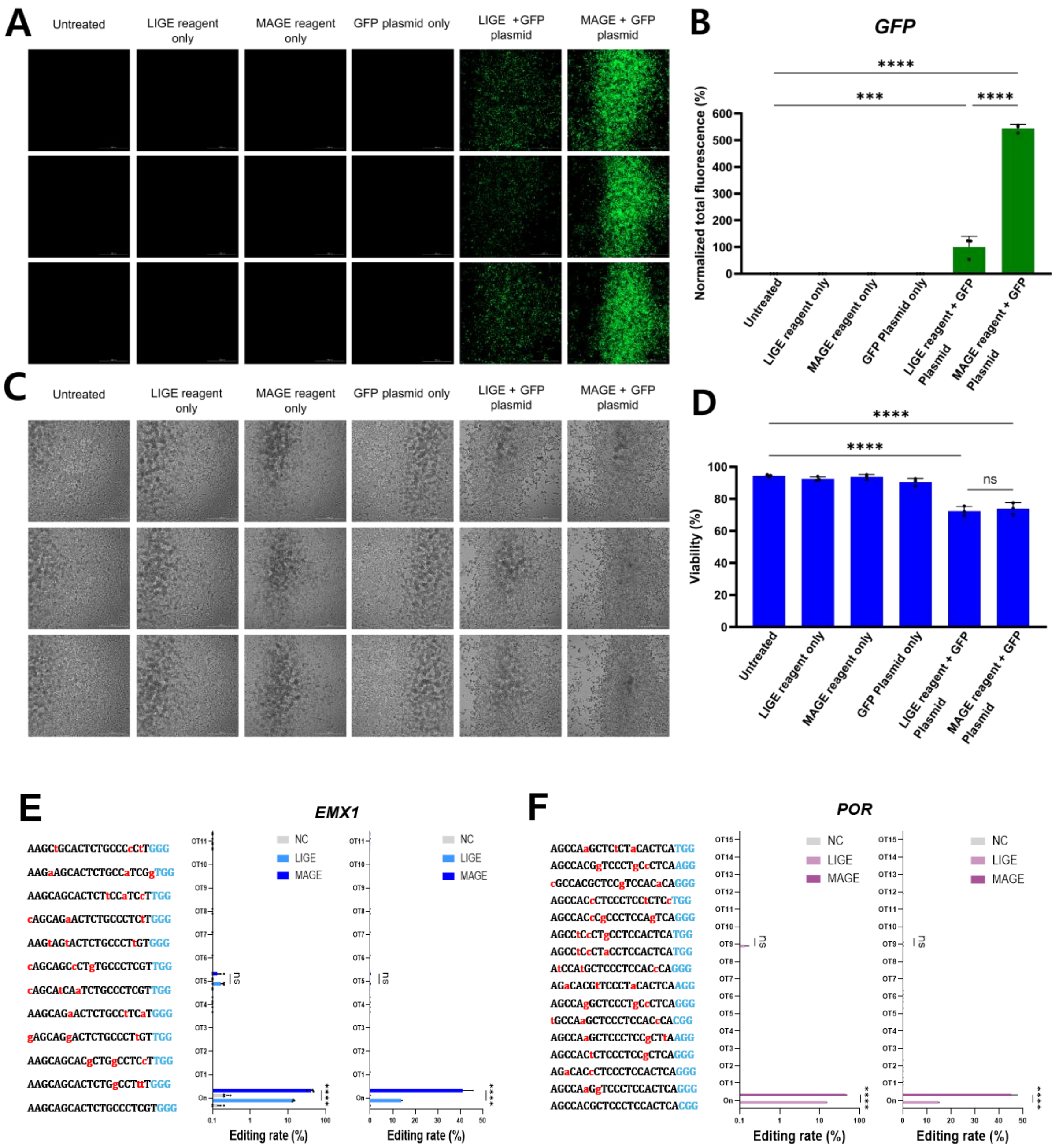

Supplemental Figure S3. Comparison of transfection efficiency and cell viability in HEK293FT cells following plasmid delivery via lipofection (LIGE) and magnetofection (MAGE). (A) Representative GFP fluorescence images of HEK293FT cells 48 h after transfection with the GFP-expressing CRISPR plasmid pCAG-SpCas9-GFP-U6-gRNA (Addgene plasmid #79144) using lipofection (LIGE) or Magnetofection (MAGE). Cells were assigned to six experimental groups: Untreated: nothing added as a negative control, LIGE only: only lipofectamine 3000 reagent was treated, MAGE only: only PolyMAG neo reagent was treated, GFP plasmid only: only CRISPR expression plasmid (pCAG-SpCas9-GFP-U6-gRNA) was treated, LIGE + GFP plasmid: CRISPR expression plasmid was co-treated with lipofectamine 3000 reagent, and MAGE + GFP plasmid: CRISPR expression plasmid was co-treated with PolyMAG neo reagent. Reagent-only control groups treated transfection reagents

without plasmid DNA, whereas the plasmid-only group treated plasmid DNA without a delivery reagent. Scale bars indicate 200 μm each. (B) Quantification of total GFP fluorescence intensity from (A). The total GFP fluorescence intensity of six experimental groups were measured and normalized to the mean fluorescence intensity of the LIGE + GFP plasmid group, which was set to 100%. Data are presented as mean ± standard error from three independent transfection experiments. (C) Representative bright-field images used for assessment of cell viability following plasmid delivery using lipofection (LIGE) or Magnetofection (MAGE). HEK293FT cells were treated with lipofection (LIGE) or magnetofection (MAGE) under the same conditions used for transfection experiments. Experimental groups included untreated control, LIGE only: only lipofectamine 3000 was treated, MAGE only: only PolyMAG neo was treated, GFP plasmid only: only CRISPR expression plasmid (pCAG-SpCas9-GFP-U6-gRNA) was treated, LIGE + GFP plasmid: CRISPR expression plasmid was co-treated with lipofectamine 3000, and MAGE + GFP plasmid: CRISPR expression plasmid was co-treated with PolyMAG neo. Scale bars indicate 200 μm each. (D) Cell viability (%) histograms from (C). Viability was calculated as the percentage of viable cells relative to the total cell count. Data are presented as mean ± standard error from three independent biological replicates. (E, F) In silico-based off-target analyses were conducted for the *EMX1* (E) and *POR* (F) target sites in HEK293FT cell line, followed by quantification of Indel frequencies (%) using targeted amplicon sequencing. Histograms are presented on both logarithmic (left) and linear (right) scales. The on-target and predicted off-target sequences are shown adjacent to each histogram. Mismatched nucleotides between the guide RNA and genomic target sequence are highlighted in red, and PAM sequences are highlighted in blue. Data are presented as mean ± standard error from three independent biological replicates. Statistical significance was determined using two-way ANOVA (ns: not significant, *$p \leq 0.05$, **$p \leq 0.01$, ***$p \leq 0.001$, ****$p \leq 0.0001$). LIGE: lipofection-mediated genome editing, MAGE: magnetofection-mediated genome editing.

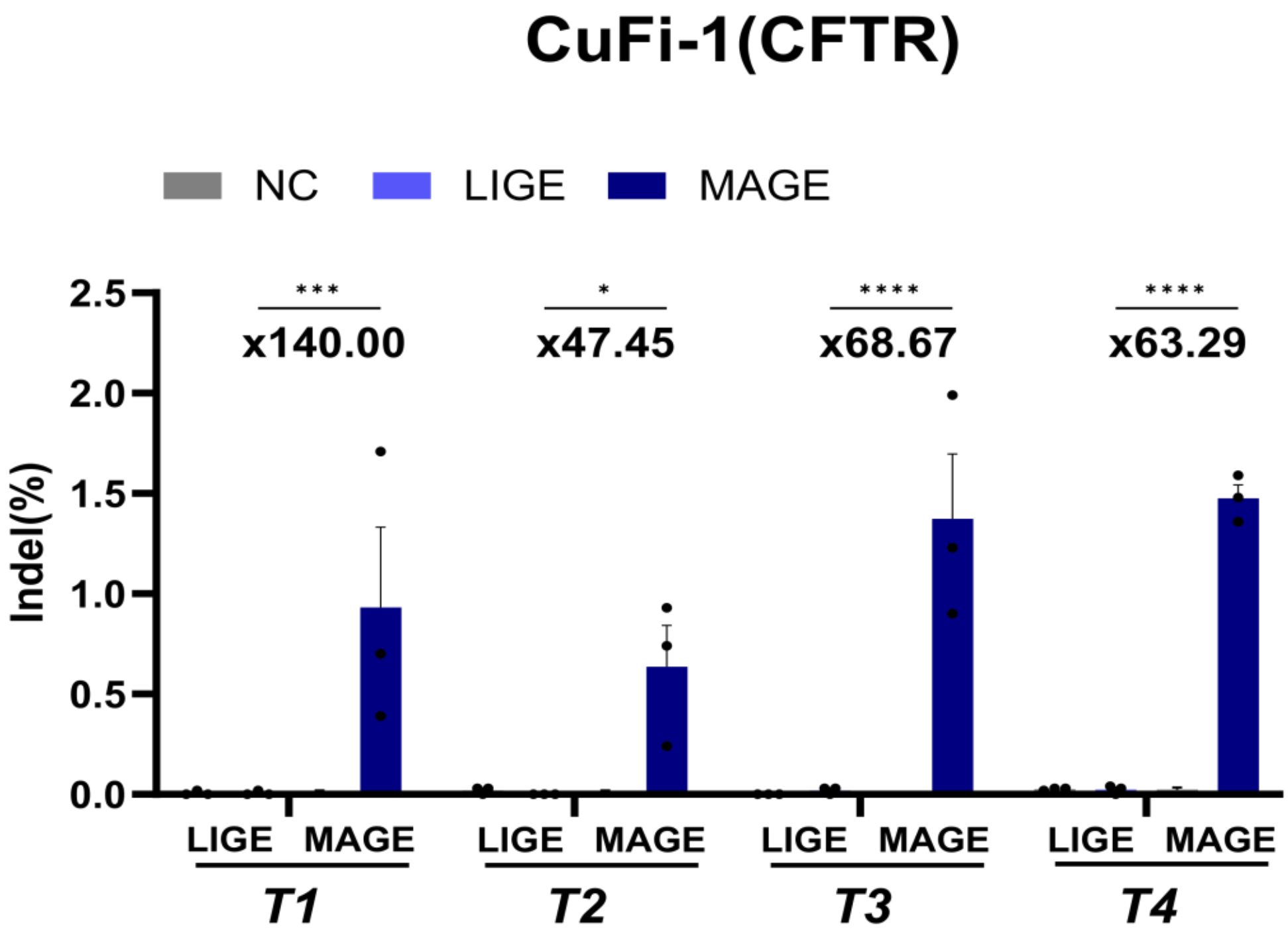

Supplemental Figure S4. Magnetic nanoparticle-mediated delivery enables highly efficient genome editing on CuFi-1 cell line. Analysis of indel induction efficiencies of SpCas9 effectors delivered *via* lipofection (LIGE) or magnetofection (MAGE) at multiple target genomic loci in the Cufi-1 cell line. Data are presented as mean ± standard error from three independent biological replicates. Statistical significance was evaluated using two-way ANOVA (ns: not significant, *$p \leq 0.05$, **$p \leq 0.01$, ***$p \leq 0.001$, ****$p \leq 0.0001$). NC: negative control, LIGE: lipofection-mediated genome editing, MAGE: magnetofection-mediated genome editing.
